# Linking Alzheimer’s Disease and Type 2 Diabetes: Characterization and Inhibition of Cytotoxic Aβ and IAPP Hetero-Aggregates

**DOI:** 10.3389/fmolb.2022.842582

**Published:** 2022-03-17

**Authors:** Kenana Al Adem, Aya Shanti, Amit Srivastava, Dirar Homouz, Sneha Ann Thomas, Mostafa Khair, Cesare Stefanini, Vincent Chan, Tae-Yeon Kim, Sungmun Lee

**Affiliations:** ^1^ Department of Biomedical Engineering and Healthcare Engineering Innovation Center, Khalifa University of Science and Technology, Abu Dhabi, United Arab Emirates; ^2^ Department of Physics, Khalifa University of Science and Technology, Abu Dhabi, United Arab Emirates; ^3^ Department of Physics, University of Houston, Houston, TX, United States; ^4^ Center for Theoretical Biological Physics, Rice University, Houston, TX, United States; ^5^ Core Technology Platforms, New York University Abu Dhabi, Abu Dhabi, United Arab Emirates; ^6^ Department of Civil Infrastructure and Environmental Engineering, Khalifa University of Science and Technology, Abu Dhabi, United Arab Emirates; ^7^ Khalifa University’s Center for Biotechnology, Khalifa University of Science and Technology, Abu Dhabi, United Arab Emirates

**Keywords:** co-aggregation, cross-interaction, cross-seeding, co-aggregation inhibition, cellular toxicity, epigallocatechin gallate, β-amyloid, islet amyloid polypeptide

## Abstract

The cytotoxic self-aggregation of β-amyloid (Aβ) peptide and islet amyloid polypeptide (IAPP) is implicated in the pathogenesis of Alzheimer’s disease (AD) and Type 2 diabetes (T2D), respectively. Increasing evidence, particularly the co-deposition of Aβ and IAPP in both brain and pancreatic tissues, suggests that Aβ and IAPP cross-interaction may be responsible for a pathological link between AD and T2D. Here, we examined the nature of IAPP-Aβ40 co-aggregation and its inhibition by small molecules. In specific, we characterized the kinetic profiles, morphologies, secondary structures and toxicities of IAPP-Aβ40 hetero-assemblies and compared them to those formed by their homo-assemblies. We demonstrated that monomeric IAPP and Aβ40 form stable hetero-dimers and hetero-assemblies that further aggregate into β-sheet-rich hetero-aggregates that are toxic (cell viability <50%) to both PC-12 cells, a neuronal cell model, and RIN-m5F cells, a pancreatic cell model for β-cells. We then selected polyphenolic candidates to inhibit IAPP or Aβ40 self-aggregation and examined the inhibitory effect of the most potent candidate on IAPP-Aβ40 co-aggregation. We demonstrated that epigallocatechin gallate (EGCG) form inter-molecular hydrogen bonds with each of IAPP and Aβ40. We also showed that EGCG reduced hetero-aggregate formation and resulted in lower β-sheets content and higher unordered structures in IAPP-Aβ40-EGCG samples. Importantly, we showed that EGCG is highly effective in reducing the toxicity of IAPP-Aβ40 hetero-aggregates on both cell models, specifically at concentrations that are equivalent to or are 2.5-fold higher than the mixed peptide concentrations. To the best of our knowledge, this is the first study to report the inhibition of IAPP-Aβ40 co-aggregation by small molecules. We conclude that EGCG is a promising candidate to prevent co-aggregation and cytotoxicity of IAPP-Aβ40, which in turn, contribute to the pathological link between AD and T2D.

## Introduction

Alzheimer’s disease (AD) and type two diabetes (T2D) are highly prevalent aging-related disorders with substantial economic, social and health burdens (Prince et al., 2016; Saeedi et al., 2019). AD constitutes the most common forms (60–70%) of dementia which in turn, is expected to affect around 131.5 million individuals worldwide by 2050 (Prince et al., 2016). In addition, T2D accounts for the majority of diabetic cases (90%) and is expected to affect 700 million individuals by 2045 (Saeedi et al., 2019). Ongoing research has established a strong association between AD and T2D in which patients of T2D were shown to be at a higher risk of developing AD (Cukierman et al., 2005; Biessels et al., 2006; Vagelatos and Eslick, 2013; Beeri and Bendlin, 2020) and vice versa (Janson et al., 2004). In fact, T2D patients are estimated to be 1.46 times more likely to develop AD compared to non-diabetic subjects (Cheng G et al., 2012). Various epidemiological (Zhong et al., 2012; Ekblad et al., 2017; Kong et al., 2018), *in vivo* (Li et al., 2007; Wang Y. et al., 2014; Mehla et al., 2014; Zhang et al., 2017) and *in vitro* studies (Xie et al., 2002; Zhao et al., 2008; Gali et al., 2019) suggest insulin resistance and glucose imbalance as the underlying physiological mechanisms that link AD and T2D. However, recent work suggests that the link between the two diseases can be addressed at an even smaller level, particularly, at the protein level, where the co-deposition of amyloids and the cross-interaction of amyloid-generating peptides can mediate the cross-talk between AD and T2D (Morales et al., 2009, 2013; Miklossy et al., 2010; Jackson et al., 2013; Martinez-Valbuena et al., 2019, 2021; Raimundo et al., 2020; Stanciu et al., 2020; Menezes et al., 2021; Zhang et al., 2021).

Linking AD and T2D from the protein aggregation perspective stems from the amyloid-like characteristics of both diseases which are marked by the excessive cell loss due to the deposition of β-amyloid (Aβ) aggregates and the islet amyloid polypeptide (IAPP) aggregates in neuronal and pancreatic tissues, respectively (Murphy and LeVine, 2010; Jurgens et al., 2011; Chiti and Dobson, 2017). The cytotoxicity induced by the aggregation of intrinsically disordered proteins such as Aβ and IAPP have been shown to be linked to membrane damage (Brender et al., 2012; Sciacca et al., 2020). Recently, the lipid-chaperone hypothesis was proposed to describe the complex mechanism by which intrinsically disordered proteins cause plasma membrane damage through lipid interactions (Sciacca et al., 2020). Intrinsically disordered proteins were shown to interact with free lipids to form lipid-protein complexes that further facilitate protein insertion into plasma membrane through ion-channel-like or detergent-like approaches depending on the critical micellar concentration of the membrane (La Rosa et al., 2016; Scollo et al., 2018; Sciacca et al., 2020; Scollo and La Rosa, 2020). Hence, the lipid-chaperone hypothesis describes the mechanism by which both amyloids and oligomers cause membrane damage and eventually cellular loss.

Although IAPP (also known as Amylin) is a pancreatic-derived peptide whereas Aβ is a brain-derived peptide, multiple lines of evidence suggest their cross-interaction and co-deposition both in the brain and in the pancreas (Zhang et al., 2021). In fact, Aβ40/42 and hyper-phosphorylated tau deposits were found in the pancreatic tissues of T2D patients where Aβ deposits were co-localized with IAPP in the affected islets of Langerhans (Miklossy et al., 2010; Martinez-Valbuena et al., 2019; Martinez-Valbuena et al., 2021).

Similarly, IAPP was found to enter the brain from circulation by crossing the blood brain barrier to reach to the parenchyma and the interstitial fluid of the cerebral cortex (Banks et al., 1995; Banks and Kastin, 1998; Jackson et al., 2013). Furthermore, IAPP was detected in the cerebrospinal fluid in the pico-molar range and its levels were elevated in AD patients compared to healthy subjects (Fawver et al., 2014). Importantly, studies have reported the co-localization of IAPP and Aβ deposits in brain tissues of AD patients with and without diabetes (Jackson et al., 2013; Oskarsson et al., 2015; Martinez-Valbuena et al., 2019; Martinez-Valbuena et al., 2021).

More recent *in vivo* and *in vitro* studies have examined the capability of IAPP and Aβ in cross-seeding each other and augmenting the amyloid formation events. Animal studies have shown that injecting IAPP and Aβ42 fibrils in mice expressing human IAPP led to enhanced IAPP deposition in the pancreatic tissues possibly through homologous and heterologous seeding mechanisms (Oskarsson et al., 2015; Wijesekara et al., 2017). In turn, IAPP aggregates in the brain of transgenic mice efficiently cross-seeded Aβ and enhanced Aβ burden as manifested by the higher number and the larger size of amyloid plaques in the hippocampus and cortex regions of treated mice as compared with the control groups (Moreno-Gonzalez et al., 2017; Wijesekara et al., 2017; Xi et al., 2019).

As for *in vitro* studies, monomeric IAPP and Aβ were shown to strongly interact with one another with binding affinities in the low nano-molar range (Andreetto et al., 2010). Although an early study (O’Nuallain et al., 2004) has shown that IAPP fibrils could not efficiently seed Aβ40 fibrillation while Aβ40 fibrils could efficiently seed IAPP fibrillation, more recent studies found that co-incubating IAPP and Aβ at the pre-fibrillar stage results in the misfolding of both peptides and the formation of hetero-fibrils through co-aggregation pathways that are characterized with distinct nucleation and elongation events in comparison to those of the self-aggregation pathways (Yan et al., 2007; Yan et al., 2014; Hu et al., 2015; Young et al., 2015; Ge et al., 2018; Bharadwaj et al., 2020; Zhang et al., 2021). Unlike previous studies which performed *in vitro* co-incubation of IAPP and Aβ in bulk solutions, one study has examined the cross-interaction of IAPP and Aβ40 mixture in the presence of isolated β-cell membrane lipids and found that IAPP-Aβ40 aggregated into hetero-complexes and permeabilized the membrane system at a rate that is slower than samples of pure IAPP but faster than samples of pure Aβ40 (Seeliger et al., 2012). To elucidate the observed cross-seeding efficiency of IAPP and Aβ, a study revealed, using Cryo-EM, a high structural similarity for the 11-residues fibril-forming segments of both IAPP (19–29, S20G) and Aβ (24–34) where each of these segments seeded the self-fibrillation of their parent full-length peptides (Krotee et al., 2018). Taken together, the above findings provide a strong evidence for the roles of the amyloidogenic IAPP and Aβ in linking T2D with AD at a protein level.

To date, effective disease modifying therapies to tackle protein misfolding diseases are not yet clinically implemented. Ongoing research attempts to develop such solutions by inhibiting the aggregation of pathogenic proteins and ameliorating their cytotoxic effects (Chiti and Dobson, 2017). Natural small molecules are among the most investigated protein aggregation inhibitors (DeToma et al., 2012; Stefani and Rigacci, 2013; Longhena et al., 2017). However, existing literature using small molecule inhibitors has mainly focused on the prevention of fibril formation of a single peptide only (i.e., IAPP alone or Aβ alone). To the best of our knowledge, no study has yet investigated the inhibition of hetero-aggregates formed by the cross-interaction of IAPP and Aβ using small molecules. As mentioned above, the cross-interaction and co-deposition of IAPP and Aβ were shown by numerous histological, *in vivo* animal models and *in vitro* studies to contribute to the pathological link between T2D and AD at the protein level; hence, preventing IAPP and Aβ cross-interaction, in addition to self-interaction, is a promising therapeutic strategy to address T2D-associated AD and vice versa. Therefore, this study aims to: 1) Characterize the hetero-aggregates formed *via* the cross-interaction of IAPP and Aβ40 in comparison to those formed *via* IAPP and Aβ40 self-interactions in terms of kinetic aggregation pathways, secondary structure and morphological changes as well as cytotoxic effects on neuronal and pancreatic cell models; 2) Investigate the most potent candidate inhibitor, out of 6 selected polyphenolic candidates, in preventing the self-aggregation of IAPP and Aβ40; 3) Test the ability of the most potent inhibitor in preventing IAPP-Aβ40 co-aggregation by assessing its inhibitory effect on the co-aggregation kinetic pathway, morphologies, secondary structure as well as its role in preventing the hetero-aggregate cytotoxic effects on neuronal and pancreatic cell models.

## Materials and Methods

### Peptides and Chemicals

Human amidated IAPP (1–37) and human Aβ (1–40) were purchased from AnaSpec (Fremont, California, United States). Human amidated IAPP (1–37) and human Aβ (1–40) purities were >96% as reported by the manufacturer AnaSpec. 1,1,1,3,3,3-Hexafluoro-2-propanol (HFIP), dimethyl sulfoxide, sodium phosphate monobasic, thioflavine-T, thiazolyl blue tetrazolium bromide, ammonium molybdate tetrahydrate, caffeic acid, curcumin, (−)-epigallocatechin gallate, silibinin (A and B diastereomers), rosmarinic acid, and myricetin were all purchased from Sigma-Aldrich (Saint Louis, MO, United States).

### Sample Preparation of Peptides and Inhibitors

IAPP or Aβ40 stock solutions were prepared by incubating each peptide in 100% HFIP at 2.56 mM for 8 h at room temperature. HFIP is a well-known fluorinated alcohol that is highly polar and has strong hydrogen binding properties that induces the monomeric and un-aggregated conformations of the peptides; hence, the peptides were kept in HFIP until the beginning of the experiment to ensure the removal of pre-existing aggregates and to maintain the peptides in monomeric conformations (Kayed et al., 1999; Krampert et al., 2000; Stine et al., 2003; Yan et al., 2006; Yan et al., 2007; Andreetto et al., 2010; Yan et al., 2014). At the beginning of the experiments, the stock solutions of IAPP or Aβ40 in HFIP were diluted to the required concentrations in phosphate buffer (10 mM NaH_2_PO_4_, pH 7.4) with a final HFIP ≤1% (v/v). Keeping residual amounts of HFIP (<1%) in the buffer system was shown by previous studies (Kayed et al., 1999; Krampert et al., 2000; Stine et al., 2003; Yan et al., 2006; Yan et al., 2007; Andreetto et al., 2010; Yan et al., 2014) to ensure high reproducibility of the self-assembly results. Due to the high aggregation propensity of IAPP and Aβ40 and to keep them in non-aggregated states, the peptides HFIP stock solutions were diluted in ice-cold buffer systems. In all experiments, the phosphate buffer (10 mM NaH_2_PO_4_, pH 7.4) was autoclaved and double filtered using syringe-filters prior to each experiment.

Samples containing IAPP alone or Aβ40 alone, at 10, 20 and 40 μM, were prepared by diluting required volumes of IAPP or Aβ40 HFIP stocks in phosphate buffer (10 mM NaH_2_PO_4_, pH 7.4) with HFIP ≤1% (v/v). The mixed samples of IAPP-Aβ40 were prepared by co-incubating monomeric IAPP and monomeric Aβ40 at equimolar concentration 10 µM:10 µM or 20 µM:20 µM of each peptide) by diluting the required volumes of IAPP and Aβ40 HFIP stocks in phosphate buffer (10 mM NaH_2_PO_4_, pH 7.4) with HFIP ≤1% (v/v).

For the inhibition of co-aggregation experiments, IAPP-Aβ40 mixed samples were prepared by co-incubating monomeric IAPP and monomeric Aβ40 at equimolar concentration (20 µM:20 µM) in the presence of increasing EGCG concentrations, 10, 20, 40, and 100 µM.

For the inhibitors screening tests, stock solutions of the candidate inhibitors, caffeic acid, (−)-epigallocatechin gallate (EGCG), myricetin, silibinin, curcumin and rosmarinic acid, were freshly prepared prior to each experiment. For caffeic acid, myricetin, silibinin, curcumin, and rosmarinic acid, stock solutions were prepared in 100% DMSO before further dilution in phosphate buffer (10 mM NaH_2_PO_4_, pH 7.4) with a final DMSO ratio of 0.25% or 0.5% (v/v). IAPP or Aβ40 samples were individually co-incubated with each inhibitor at an equimolar concentration of 40 µM. Control samples included the incubation of IAPP alone (40 µM) or Aβ40 (40 µM) alone in the absence of the inhibitors. Also, for inhibitors which were dissolved in DMSO, control samples of IAPP alone or Aβ40 alone containing matching DMSO ratios were prepared.

To initiate the aggregation process, samples described above were incubated at 37°C under quiescent conditions and at the indicated time-points during the aggregation process, aliquots from each sample were withdrawn for the different characterization methods including the thioflavin-T (ThT) fluorescence assay, circular dichroism (CD), scanning transmission electron microscopy (STEM) and cell viability assays.

### Thioflavin-T Fluorescence Assay

**FIGURE 1:**
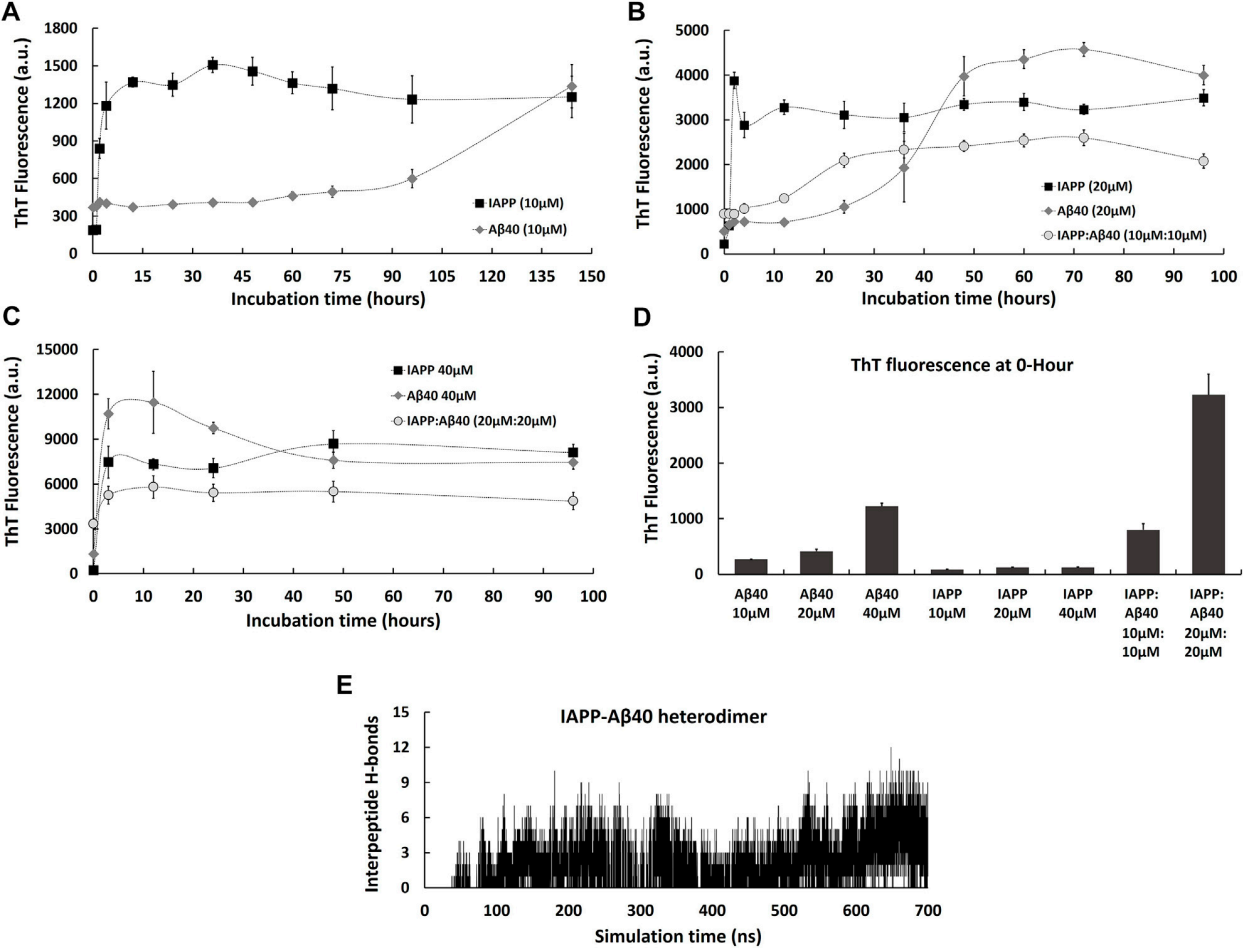
Kinetic profiles of the co-aggregation (IAPP-Aβ40 equimolar mixture) and self-aggregation (IAPP alone and Aβ40 alone) pathways. The self-aggregation profiles of IAPP alone and Aβ40 alone were acquired at three increasing concentrations: **(A)** 10 µM, **(B)** 20 µM and **(C)** 40 µM. The co-aggregation profiles of equimolar mixture of IAPP:Aβ40 were acquired at **(B)** 10 µM:10 µM and **(C)** 20 µM:20 µM. **(D)** ThT fluorescence intensities of the freshly prepared samples (i.e., 0-Hour) of IAPP alone, Aβ40 alone and IAPP-Aβ40 at all examined concentrations. **(E)** Time evolution of inter-peptide hydrogen bonds formed between IAPP and Aβ40 in their hetero-dimer system. All ThT fluorescence data (a.u.) represent mean ± SE (*n* = 3).

Thioflavin-T (ThT) stock solution was freshly prepared prior to each experiment by dissolving the ThT dye in autoclaved Milli-Q water at a concentration of 30 µM. The aggregation kinetic profiles of all samples described above were obtained using ThT fluorescence measurements at specific time-points during the aggregation process. At each time-point, peptide aliquots (70 µL) were mixed with ThT stock solution (70 µL) in black flat-bottom 96 well-plates (Nunclon Delta-Treated, Thermo Fisher Scientific). Immediately after mixing, ThT fluorescence intensities (λex 440 nm and λem 485 nm) were measured *via* an Infinite 200 Pro microplate reader (Tecan Trading AG, Switzerland). For each experiment, the ThT signal intensities represent the mean of at least triplicate measurements. The fluorescence value of the blank containing ThT only was measured and included in the calculations to produce all ThT data in Figure 1 and Figure 6A.

For the ThT-screening experiments of candidate polyphenolic inhibitors, additional control experiments were performed (experimental design was adopted from the study by (Hudson et al., 2009) to test whether the selected polyphenols interact with the ThT dye and interfere with its fluorescence spectra and/or competitively bind with its fibril binding sites. In the control experiments, the ThT fluorescence spectra (λex 440 nm, λem 470–700 nm) were acquired for pre-formed IAPP fibrils (40 µM) or Aβ40 fibrils (40 µM) in the presence of ThT (30 µM). Next, each polyphenol (40 µM) was added to the mixture of ThT (30 µM) and pre-formed IAPP fibrils (40 µM) or Aβ40 fibrils (40 µM) and the fluorescence spectra (λex 440 nm, λem 470–700 nm) were reacquired immediately to examine any changes in the ThT fluorescence spectra in very short periods of time.

For the inhibition of co-aggregation kinetic experiments, similar control experiments were acquired where EGCG (0.1–100 µM) was added to the mixture of ThT (30 µM) with preformed IAPP-Aβ40 hetero-aggregates to check if EGCG affects their ThT fluorescence spectra.

### Scanning Transmission Electron Microscopy

At different time-points during the aggregation process, 15 µL of peptide samples was withdrawn and placed on carbon-coated copper grids (Ted Pella Inc., CA, United States) for 2 min. Next, filter paper was utilized to absorb the remaining solution. The staining solution (15 µL), containing 2 mg/ml of ammonium molybdate, was placed on the grid for 1 min. Similarly, the remaining solution was removed by a filter paper. Images were acquired using FEI TecnaiTM T20 TEM 200 kV (Thermo Fisher Scientific, OR, United States) using the Scanning Transmission Electron Microscopy (STEM) mode as it enables the imaging of aggregates with enhanced contrast due to its HAADF (high-angle annular dark-field) detector. The obtained images were further analyzed using ImageJ software (1.52a) by measuring the dimensions of the formed assemblies (diameters of fibrillar or non-fibrillar aggregates).

### Far-UV Circular Dichroism Spectroscopy

Circular dichroism (CD) spectra were obtained for samples containing IAPP alone (10, 20 µM), Aβ40 alone (10, 20 µM) and equimolar mixture of IAPP-Aβ40 (10 µM:10 µM or 20M:20 µM). Also, samples were prepared for equimolar mixture of IAPP-Aβ40 in the absence and presence of increasing EGCG concentrations (10, 20, 40, and 100 µM). Similar to the kinetics study, all samples were prepared in phosphate buffer (10 mM NaH_2_PO_4_, pH 7.4) with 1% HFIP (v/v). For fresh (0-Hour) samples, CD measurements were obtained immediately after diluting the peptides from their HFIP stocks into the buffer. CD spectra were collected between 190 and 260 nm at room temperature at 1 nm/s scan rate in 1 mm quartz cuvettes using Chirascan CD spectrometer (Applied Photophysics). CD spectra of the buffer alone or buffer containing different EGCG concentrations were subtracted from their corresponding peptide samples to examine the secondary structural changes of the peptides only. The deconvolution of CD spectra were analyzed using DICHROWEB (Whitmore and Wallace, 2008) by selecting CONTIN as the analysis program (van Stokkum et al., 1990) and Set 4 as the reference set (Sreerama et al., 2000).

### Cell Culture

PC-12 cells (ATCC), rat pheochromocytoma-derived adrenal medulla cells, and RIN-m5F (ATCC), rat insulinoma-derived pancreatic beta-cells, were utilized in this study as the neuronal and pancreatic cell models, respectively. The base medium for both cell lines was the Roswell Park Memorial Institute (RPMI)-1640 base medium (Gibco, Thermo Fisher Scientific) containing 4.5 g/L glucose, 1.5 g/L sodium bicarbonate, 1 mM sodium pyruvate, 10 mM HEPES and 2 mM l-glutamine. For PC-12 cells, the base medium was supplemented with 10% heat-inactivated horse serum (Gibco), 5% fetal bovine serum (Gibco) and 1% penicillin-streptomycin (Gibco) whereas for RIN-m5F cells, the base medium was supplemented with 10% fetal bovine serum and 1% penicillin-streptomycin. Both cell types were maintained in a humidified incubator (37°C, 5% CO_2_).

### MTT Cell Viability Assay

Individual peptide samples including IAPP alone (20 µM) and Aβ40 alone (20 µM) as well as mixed peptide samples (IAPP:Aβ40, 20 µM:20 µM) were prepared as described in the sample preparation section. All samples were incubated at 37°C for 3 h or 96 h to produce homo-aggregates and hetero-aggregates. It is important to note that for cell viability assays, the self-aggregation and co-aggregation samples were prepared in very low volumes of HFIP such that the final HFIP volume did not exceed 0.32% (v/v); a ratio that we initially tested and found to be non-toxic on both cell lines (PC-12 and RIN-m5F cells).

Before addition to cells, samples of homo-aggregates and hetero-aggregates were diluted in serum-free base media. Homo-aggregates (IAPP alone or Aβ40 alone) were added to each cell model at increasing concentrations of 10, 100, 500 nM, 1 μ, 2 , 4 , 6, and 8 µM. Hetero-aggregates (equimolar mixture of IAPP and Aβ40) were added to each cell model at the above concentration range where each concentration represents the total concentration of both peptides (i.e., each peptide was present at 5, 50, 250 nM, 0.5, 1, 2, 3, and 4 µM).

For testing the effect of EGCG in inhibiting the cytotoxicity of hetero-aggregates, samples of IAPP-Aβ40 (20 µM:20 µM) in the absence and presence of increasing EGCG concentrations (10, 20, 40, and 100 µM) were prepared as described previously and were extracted at 96 h for the MTT experiments. IAPP-Aβ40 samples in the presence of EGCG were diluted in serum-free base media to yield final EGCG concentrations as follows: IAPP:Aβ40:EGCG at 1 µM:1 µM:5 µM, 1 µM:1 µM:2 µM, 1 µM:1 µM:1 and 1 µM:1 µM:0.5 µM. In addition, higher concentrations of the samples were also tested as follows: IAPP:Aβ40:EGCG at 2 µM:2 µM:10 µM, 2 µM:2 µM:4 µM, 2 µM:2 µM:2 µM and 2 µM:2 µM:1 µM.

Cells were seeded in clear flat-bottom 96 well-plates (Nunclon Delta-Treated, Thermo Scientific™) at 30,000 cells/well and 50,000 cells/well for RIN-m5F cells and PC-12 cells, respectively, and seeded cells were allowed to stabilize for 24 h in a humidified incubator (37°C, 5% CO_2_) before adding the treatments. Next, the treatments described above were added to each cell line and cells were incubated for 24 h under treatment in a humidified incubator (37°C, 5% CO_2_). Control cells included PC-12 or RIN-m5F cells supplemented with base media only which had matching buffer concentrations (v/v) as in the peptide treatments. After 24 h, MTT (3-(4,5-dimethylthiazol-2-yl)-2,5-diphenyl tetrazolium bromide) solution (5 mg/ml) was added to each well at a final concentration of 0.45 mg/ml and cells were incubated with MTT for an additional 4 h in a humidified incubator (37°C, 5% CO_2_). Next, supernatant was removed carefully and 100 µL of DMSO was added and mixed thoroughly in each well to dissolve the formed formazan crystals. The absorbance signal was measured at 570 nm using the Infinite 200 Pro microplate reader (Tecan Trading AG, Switzerland). Cell viability rates were obtained by comparing the absorbance of treated cells to that of control cells as follows: Cell viability rate = (treated cells with MTT)/(control cells with MTT) × 100. For each experiment, cell viability rates represent the mean of at least triplicate measurements.

In addition, to test whether EGCG interferes with MTT absorbance, wells having EGCG (0.5–40 µM) and MTT only (i.e., no cells) were regarded as the negative control and their absorbance values (at 570 nm) were obtained and later subtracted (in case of any detected interference effect) from cells treated with matching EGCG concentrations. In addition, MTT assay was used to measure the cell viability of each cell line treated with EGCG (0.5–40 µM) alone (i.e., without peptide addition) to examine whether EGCG could have its own effects on the cell viabilities.

### Live/Dead Cell Viability Assay

The LIVE/DEAD™ Cell Imaging Kit (Molecular Probes™, Thermo Fisher Scientific) was used to assess the viability of PC-12 cells and RIN-m5F cells, using Calcein AM and BOBO-3 Iodide to stain for live and dead (membrane-compromised) cells, respectively. Cells were seeded in clear flat-bottom 96 well-plates (Nunclon Delta-Treated, Thermo Scientific™) at 30,000 cells/well and 50,000 cells/well for RIN-m5F cells and PC-12 cells, respectively, and seeded cells were allowed to stabilize for 24 h in a humidified incubator (37°C, 5% CO2) before adding the treatments. Next, the cells were treated with 96 h-aged hetero-aggregates of IAPP-Aβ40 (1 µM:1 µM) in the absence and presence of EGCG at 2 µM or 5 µM. Cell treated with base media were considered as the positive control (live cells) whereas cells treated with 50% ethanol were considered as the negative control (dead cells). Cells were incubated for 24 h under treatment which was followed by the removal of the treatment and the addition of the two staining fluorophores. Fluorescence microscope (Zeiss ZEN, Germany) was used to obtain the fluorescence images of cells. Cell viability rates were calculated as follows: (number of live cells/numbers of live cells + number of dead cells) × 100. ImageJ software (1.52a) was used to count the number of live and dead cells.

### Molecular Dynamics Simulations: Systems and Conditions

Molecular dynamics (MD) simulations were performed to investigate the early stages of IAPP and Aβ40 cross-interaction by studying the formation of IAPP-Aβ40 hetero-dimers. In addition, the formation of IAPP-Aβ40 hetero-dimer in presence of EGCG was investigated to understand, at an atomic level, the inhibitory role of EGCG against IAPP-Aβ40 cross-interaction.

Two systems were modelled, the first contains one Aβ40 monomer and one IAPP monomer and the second contains one Aβ40 monomer, one IAPP monomer and 5 EGCG molecules. The starting structures of Aβ40 (PDBID: 2LFM) (Vivekanandan et al., 2011) and IAPP (PDBID: 2L86) (Nanga et al., 2011) were obtained from the Protein Data Bank. EGCG structure was built from its canonical SMILES obtained from PubChem database. Since Aβ40 and IAPP structures were resolved in multimeric forms, therefore, in this study we have only taken one monomeric structure for each peptide. The initial Aβ40 and IAPP monomeric structures for dimer simulations were taken from the most populated clusters from a preceding 500 ns MD simulation of monomeric Aβ40 and IAPP in solution.

To study the underlying mechanism of hetero-dimer formation, we placed the two monomers that correspond to Aβ40 and IAPP peptides randomly using the PACKMOL (Martínez et al., 2009). The two monomers were placed with at least 1.2 nm distance between them in a simulation box of size ∼10 nm × 10 nm × 10 nm.

All-atom MD simulations were performed using the GROMACS 2020 (Hess et al., 2008) package. All the simulations were performed using the CHARMM36m (Huang et al., 2017) with the TIP3P (Jorgensen et al., 1983) water model. To mimic the experimental condition, we added 0.01 M NaCl to the system. The equations of motions were integrated with a time-step of 2 fs. Constant temperature and pressure ensembles were used. The temperature was set to 310 K using the Berendsen thermostat. Pressure was kept constant at 1 bar using the Parrinello-Rahman barostat (Parrinello and Rahman, 1981). Periodic boundary conditions were implemented in all directions. Non-bonded interactions were truncated after 10 Å with a dispersion correction. The neighbor list for non-bonded pairs was updated with every 40 steps. We used a cut-off radius of 10 Å for the neighbor search. Long-range electrostatic interactions were computed by particle mesh Ewald summation method (Darden et al., 1993) with a grid spacing of 0.16 nm and an interpolation of order 4. Covalent bonds of water and protein were constrained to their equilibrium geometries using SETTLE (Miyamoto and Kollman, 1992) and LINCS (Hess et al., 1997) algorithms, respectively. Data was recorded every 2 ps for further analysis.

For the hetero-dimer systems, we employed the first minimization run of 8,000 steps to remove the bad contacts that may arise due to the random placement of water and ions. The equilibration was performed with a positional restraint under NVT ensemble at 310 K for 10 ns. Then, NPT ensemble simulations were performed for 10 ns at 1 bar. Finally, the NPT production run was performed for 700 ns at 310 K and 1 bar without any restraint.

VMD (Humphrey et al., 1996) was used to analyze the MD trajectories by identifying the inter-peptide hydrogen bonds that form at the dimer interface between IAPP chain and Aβ40 chain. Hydrogen bonds were considered when the distance between acceptor and donor atoms is below 3.0 Å and when the angle between donor-Hydrogen-acceptor is less than 20°.

The secondary structure of each Aβ40-IAPP residue was determined using the defined secondary structure program (DSSP), invoked *via* the GROMACS tool do_dssp (Zhang and Sagui, 2015). To facilitate a clear representation, the data of similar secondary structure were grouped together: β-strands and β-bridges were coupled as β-sheets.

### Statistical Analysis

Data were presented as the mean ± standard error (SE) for n independent trials as reported in the figure captions. Unpaired t test was used to compare between control and treated samples. In all statistical analysis, *p*-values < 0.05 were considered significant and were reported in the figure captions.

## Results

### Characterizing the Kinetic Profiles of IAPP-Aβ40 Co-Aggregation vs. Self-Aggression

To better understand the differences between the co-aggregation and self-aggregation processes of IAPP and Aβ, and to examine how self-interaction and cross-interaction affect the total amount of aggregate formation, we utilized the ThT fluorescence assay to characterize the self-aggregation (IAPP alone and Aβ40 alone) and co-aggregation (IAPP-Aβ40 mixture) kinetic profiles as demonstrated in Figure 1. In addition, Figure 1 reports the cross-interaction of IAPP and Aβ40 that was investigated using molecular dynamics (MD) simulations of the hetero-dimer formation of IAPP-Aβ40.

We started by characterizing IAPP and Aβ40 self-aggregation kinetics at three increasing concentrations (10, 20, and 40 µM). As seen in Figure 1A, IAPP (10 µM) showed a self-aggregation process that is marked with a fast nucleation event (1 h only), a high elongation rate and an early occurrence of the saturation phase indicating a highly aggregation-prone peptide. However, Aβ40 (10 µM) showed a noticeably slower self-aggregation process that is marked with a well-defined nucleation period of 72 h followed by an elongation phase between 72–144 h. As shown in Figure 1B, increasing Aβ40 concentration from 10 to 20 µM resulted in reducing the nucleation event from 72 to 24 h. In addition, increasing Aβ40 concentration to 40 µM resulted in diminishing the nucleation time as seen in Figure 1C. Similarly, increasing IAPP concentration from 10 to 20 , and 40 µM completely diminished the nucleation time and dramatically increased the ThT fluorescence intensities over the course of the experiment (Figures 1B,C).

Next, we characterized co-aggregation kinetics of IAPP-Aβ40 samples, prepared by co-incubating equimolar concentrations of monomeric IAPP and Aβ40, at two increasing concentrations (IAPP:Aβ40, 10 µM:10 µM and 20 µM:20 µM). As seen in Figure 1B, the co-aggregation pathway of IAPP:Aβ40 (10 µM:10 µM) had a nucleation period of 12 h, which is substantially longer than that of IAPP alone (20 µM) and shorter than that of Aβ40 alone (20 µM). However, IAPP:Aβ40 sample at a higher concentration of each peptide (20 µM:20 µM) did not show a nucleation time; an observation that was also seen in the individual peptide samples at equivalent concentrations, IAPP (40 µM) and Aβ40 (40 µM), which also lacked well-defined nucleation periods. In fact, the absence of nucleation events in these samples is likely due to the high concentration (40 µM) employed, which led to strong and immediate interactions of IAPP and/or Aβ40 monomers and the instant formation of early aggregates. Some previous studies (Yan et al., 2007; Yan et al., 2014; Hu et al., 2015) reported elongated nucleation periods in IAPP-Aβ40 co-aggregation in comparison to self-aggregation, while others (Young et al., 2015; Ge et al., 2018) reported intermediate nucleation periods of IAPP-Aβ40 that were close to that of IAPP alone or Aβ40 alone. In our work, we tested two IAPP-Aβ40 concentrations and we observed an intermediate nucleation event for IAPP:Aβ40 (10 µM:10 µM), while no nucleation event was detected for IAPP:Aβ40 (20 µM:20 µM).

To examine the difference between the amount of homo-aggregate and hetero-aggregate formation at the end of the incubation periods, we compared the final ThT fluorescence intensities of IAPP-Aβ40 with those of individual peptide samples at equivalent concentrations. As seen in Figures 1B,C, the final ThT fluorescence intensity of IAPP:Aβ40 (10 µM:10 µM) is lower than that of IAPP alone (20 µM) and Aβ40 alone (20 µM). Similarly, the final ThT fluorescence intensity of IAPP:Aβ40 (20 µM:20 µM) is significantly less than that of IAPP alone (40 µM) and Aβ40 alone (40 µM). These results suggest that IAPP-Aβ40 cross-interaction reduces, although does not prevent, the hetero-aggregate formation in comparison to homo-aggregate formation.

**FIGURE 2:**
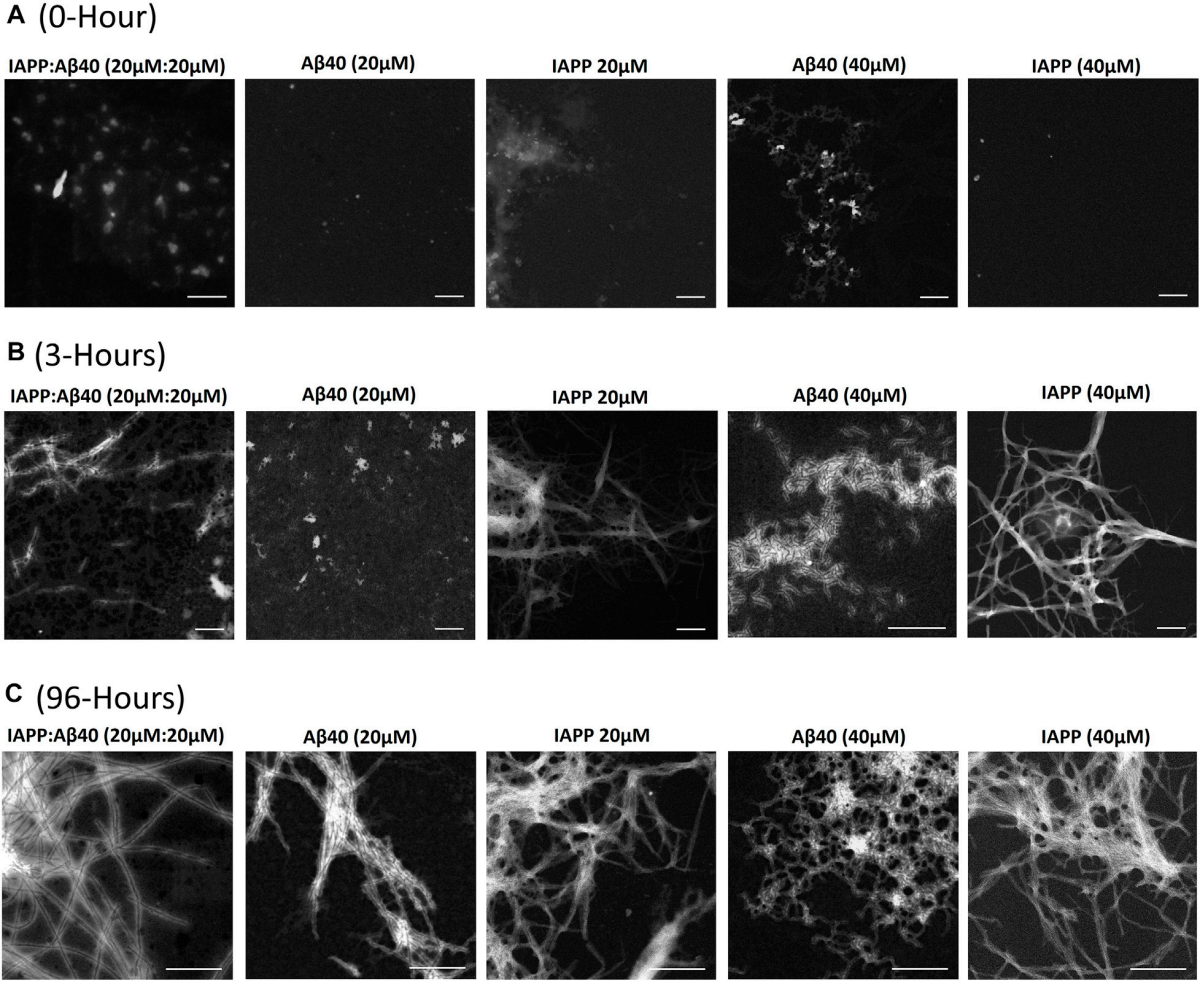
Morphological characterization using scanning transmission electron microscopy (STEM) of **(A)** 0-Hour, **(B)** 3-Hours, and **(C)** 96-Hours assemblies formed by the co-aggregation of IAPP-Aβ40 (20 µM:20 µM), as well as those formed by the self-aggregation of IAPP alone (20 and 40 µM) and Aβ40 alone (20 and 40 µM). All scale bars represent 200 nm.

The ThT data of freshly prepared samples (i.e., 0-Hour time-point) in Figure 1D reveal another distinction between the mixed and individual peptide samples. Particularly, the ThT fluorescence of freshly prepared IAPP:Aβ40 (10 µM:10 µM) is 1.7-fold and 4-fold higher than Aβ40 alone (20 µM) and IAPP alone (20 µM), respectively. Similarly, the ThT fluorescence of freshly prepared IAPP:Aβ40 (20 µM:20 µM) is 2.5-fold and 15-fold higher than Aβ40 alone (40 µM) and IAPP alone (40 µM), respectively. These results suggest that mixing monomers of IAPP and Aβ40 leads to their strong binding and the instant formation of hetero-assemblies (morphologies will be demonstrated in Figure 2A) that in turn, enhance ThT fluorescence. The strong binding between IAPP and Aβ40 can be first interpreted by their electrostatic interactions where at physiological pH, IAPP is positively charged (net charge of +3 (Akter et al., 2016) whereas Aβ40 is negatively charged (net charge of −3 (Assarsson et al., 2014; Yang et al., 2018)). Hence, monomeric IAPP and Aβ40 may exert attractive electrostatic interactions on one another when mixed together.

Next, to verify that the co-aggregation pathways presented in Figures 1B,C are mediated by hetero-assemblies that form due to IAPP and Aβ40 cross-interaction, we employed MD simulations to investigate the early events of IAPP-Aβ40 hetero-dimer formation. The formation of IAPP-Aβ40 hetero-dimer during the MD simulation was analyzed by identifying the number of inter-peptide hydrogen bonds that form at the dimer interface between chain A (IAPP) and chain B (Aβ40). As shown in Figure 1E, after 50 ns of initiating the MD simulation of IAPP and Aβ40 monomers, the number of inter-peptide hydrogen bonds started to evolve and increase with time to reach up to 12 hydrogen bonds that stabilize IAPP-Aβ40 hetero-dimer (see Supplementary Figure S1A for a representative snapshot of IAPP-Aβ40 hetero-dimer).

A previous study (Andreetto et al., 2010) has experimentally investigated the cross-interaction of IAPP and Aβ40, where monomeric IAPP and Aβ40 were shown to strongly interact with binding affinities in the low nano-molar range. In addition, the same group and *in silico* studies reported specific hot regions within IAPP and Aβ40 sequences that mediate their cross-interaction (Andreetto et al., 2010; Bakou et al., 2017; D’Urso et al., 2018; Ge et al., 2018). Hence, our results and previous studies prove the high propensity of IAPP and Aβ40 to cross-interact when mixed together to form hetero-assemblies that drive the co-aggregation pathways seen in ThT experiments (Figure 1). In fact, IAPP and Aβ40 share a high sequence similarity, including 25% sequence identity, as indicated by their sequence alignment (Supplementary Figure S2). The high similarity in their sequences, the existence of sequence-specific binding sites and their common β-sheet structural basis, provide mechanistic justifications of their co-aggregation and cross-seeding (Konstantoulea et al., 2021b; Zhang et al., 2021).

### Characterizing the Morphological Changes of IAPP-Aβ40 Co-Aggregation vs. Self-Aggregation

Scanning Transmission Electron Microscopy (STEM) was used to characterize the morphologies of samples containing IAPP alone (20 µM or 40 µM), Aβ40 alone (20 µM or 40 µM) and IAPP-Aβ40 mixture (20 µM:20 µM) at three time-points during the self- and co-aggregation pathways, 0-Hour (fresh samples), 3-Hours (early aggregates) and 96-Hours (late aggregates) as shown in Figure 2 and Supplementary Table S1.

In Figure 2A, the freshly prepared (i.e., 0-Hour) IAPP-Aβ40 sample showed hetero-assemblies that are on average larger in diameter (38.4 ± 5.4 nm) than the freshly prepared homo-assemblies of IAPP alone at 20 µM (13.5 ± 3.8 nm) and 40 µM (19.6 ± 6.2 nm) as well as Aβ40 alone at 20 µM (15.5 ± 4.7 nm) and 40 µM (24.2 ± 5.2 nm). The STEM findings suggest that the cross-interaction of IAPP and Aβ40 lead to the instant formation of large non-fibrillar hetero-assemblies that showed enhanced ThT fluorescence in comparison to homo-assemblies at the start of the ThT assay (Figure 1D). In fact, a previous study showed, using mass spectrometry, that monomeric IAPP and Aβ40 form hetero-oligomers which consist of dimers and trimers of both peptide subunits that are different from the oligomers of IAPP alone or Aβ40 alone (Young et al., 2015), while another paper (Bharadwaj et al., 2020) demonstrated that IAPP-Aβ42 samples had higher formation of high molecular weight oligomers and large aggregates in comparison to Aβ42 alone samples.

Figure 2B demonstrates the STEM images of the 3 h-aged assemblies formed in the individual and mixed peptide samples. IAPP samples (20 and 40 µM) formed intense fibrillar bundles, whereas Aβ40 (20 µM) was still in its lag phase (Figure 1B), and round structures were only observed. Although the ThT profile of Aβ40 (40 µM) showed a strong fluorescence intensity at 3-Hours (Figure 1C), STEM revealed the formation of only thin and short proto-fibril assemblies (fibril diameter 5.2 ± 1.1 nm). Importantly, the 3 h-aged IAPP-Aβ40 (20 µM:20 µM) samples formed early assemblies that were mainly populated by short fibrils (fibril diameter 12.0 ± 2.2 nm) in addition to other amorphous aggregates that were not observed in the homo-assemblies of IAPP (40 µM) or Aβ40 (40 µM); see Supplementary Figure S3 for an additional representation of the amorphous hetero-assemblies. Our findings are in line with previous studies (Yan et al., 2007; Hu et al., 2015) that depicted the formation of both fibrillar and non-fibrillar assemblies in IAPP-Aβ samples at early time-points.

Figure 2C shows that all samples (both mixed and individual peptides) formed mature fibrils as end products of their self- or co-aggregation pathways but with slight variations in the fibril diameters (see Supplementary Table S1). Our results agree with previous studies that reported the formation of mature fibrils at the end of IAPP-Aβ40/42 co-aggregation (Yan et al., 2007, 2014; Hu et al., 2015; Young et al., 2015). One study (Yan et al., 2014) further demonstrated, using double immuno-gold TEM, the presence of both peptides (IAPP and Aβ40) in the hetero-fibrils that formed in the aged IAPP-Aβ40 samples in addition to the presence of some homo-assemblies; such findings highlight that IAPP and Aβ40 cross-interaction yield hetero-fibrils containing both peptides. Another study (Bharadwaj et al., 2020), suggested the formation of amorphous IAPP-Aβ42 hetero-aggregates with thin fibrils extending along the edges of the amorphous deposits.

### Characterizing the Secondary Structure and Conformational Changes of IAPP-Aβ40 Co-Aggregation vs. Self-Aggregation

Circular dichroism (CD) was utilized to examine the secondary structure and conformational changes of samples containing IAPP alone, Aβ40 alone and IAPP-Aβ40 which enabled us to verify the formation of hetero-assemblies due to the cross-interaction between IAPP and Aβ40. The estimated secondary structural elements obtained from the deconvolution of CD spectra are shown in Supplementary Table S2.

**FIGURE 3:**
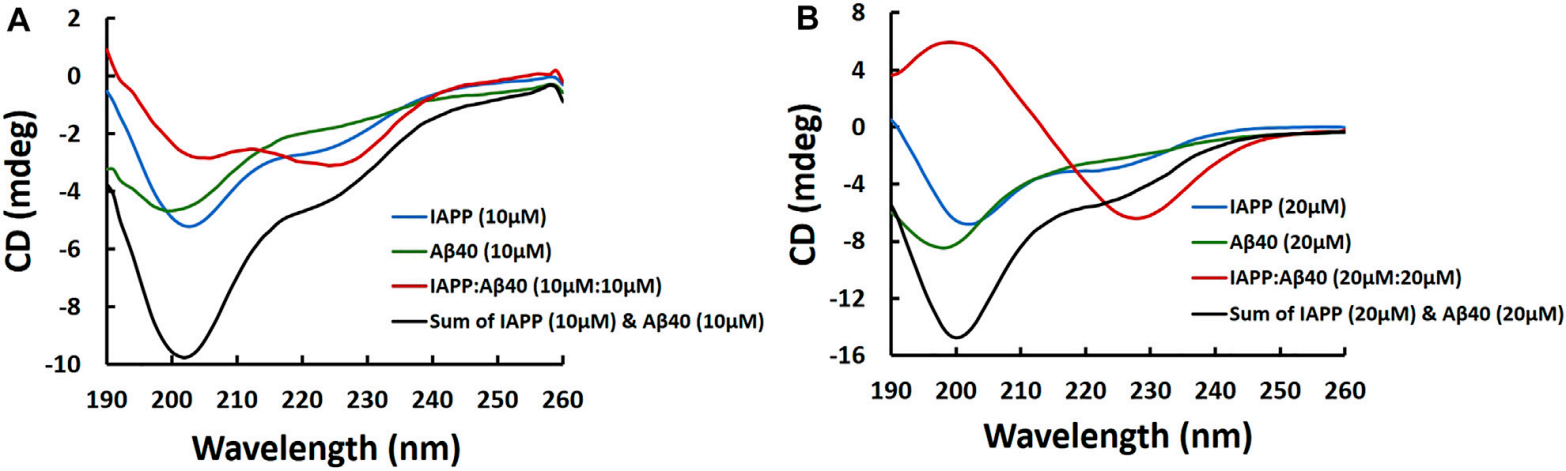
Far-UV CD spectra of samples containing IAPP alone, Aβ40 alone and IAPP-Aβ40 (equimolar mixture) prepared at increasing concentrations as shown in **(A)** and **(B)**. Fresh samples were prepared by diluting peptides from their HFIP stocks into the buffer (10 mM NaH_2_PO_4_, pH 7.4) and CD measurements were acquired immediately.

Figure 3A shows the CD spectrum of the freshly prepared IAPP-Aβ40 (10 µM:10 µM) sample which had two broad negative peaks at 205 and 226 nm, unlike the spectrum of IAPP alone (10 µM) or Aβ40 alone (10 µM) that had pronounced negative peaks at 200 nm which corresponds to their intrinsically disordered nature (Yan et al., 2007; Hu et al., 2015). This result suggests the formation of more ordered structures in the mixed sample at an early time-point (see Supplementary Table S2). Importantly, IAPP-Aβ40 (10 µM:10 µM) spectrum (red spectrum in Figure 3A) does not represent the spectral summation of IAPP alone (10 µM) and Aβ40 alone (10 µM) (black spectrum in Figure 3A) which demonstrates the occurrence of a cross-interaction event between IAPP and Aβ40 that yields hetero-assemblies with distinct CD spectra in comparison to those of the homo-assemblies. Additionally, the binding and cross-interaction of IAPP and Aβ40 was further shown in Figure 3B as the CD spectrum of IAPP-Aβ40 (20 µM:20 µM) is markedly different than the spectrum of IAPP alone (20 μM) or Aβ40 alone (20 μM) and does not represent their spectral sum. By comparing Figures 3A,B, the CD spectrum of IAPP-Aβ40 (10 µM:10 µM) lacks the positive peak at 200 nm and has less pronounced peak at 228 nm in comparison to IAPP-Aβ40 (20 µM:20 µM) indicating that the increase in peptide concentration in the mixture reduces the unordered structures and increases the β-sheets contents of the hetero-assemblies (see Supplementary Table S2).

At the end of the incubation period (i.e., 96-Hours), the CD spectra of the mixed and individual peptide samples all had negative peaks at 219–222 nm indicating the presence of either aggregated or co-aggregated β-sheets assemblies (Supplementary Figure S4 and Supplementary Table S2).

### Characterizing the Cytotoxicity of IAPP-Aβ40 Co-Aggregation vs. Self-Aggregation

**FIGURE 4:**
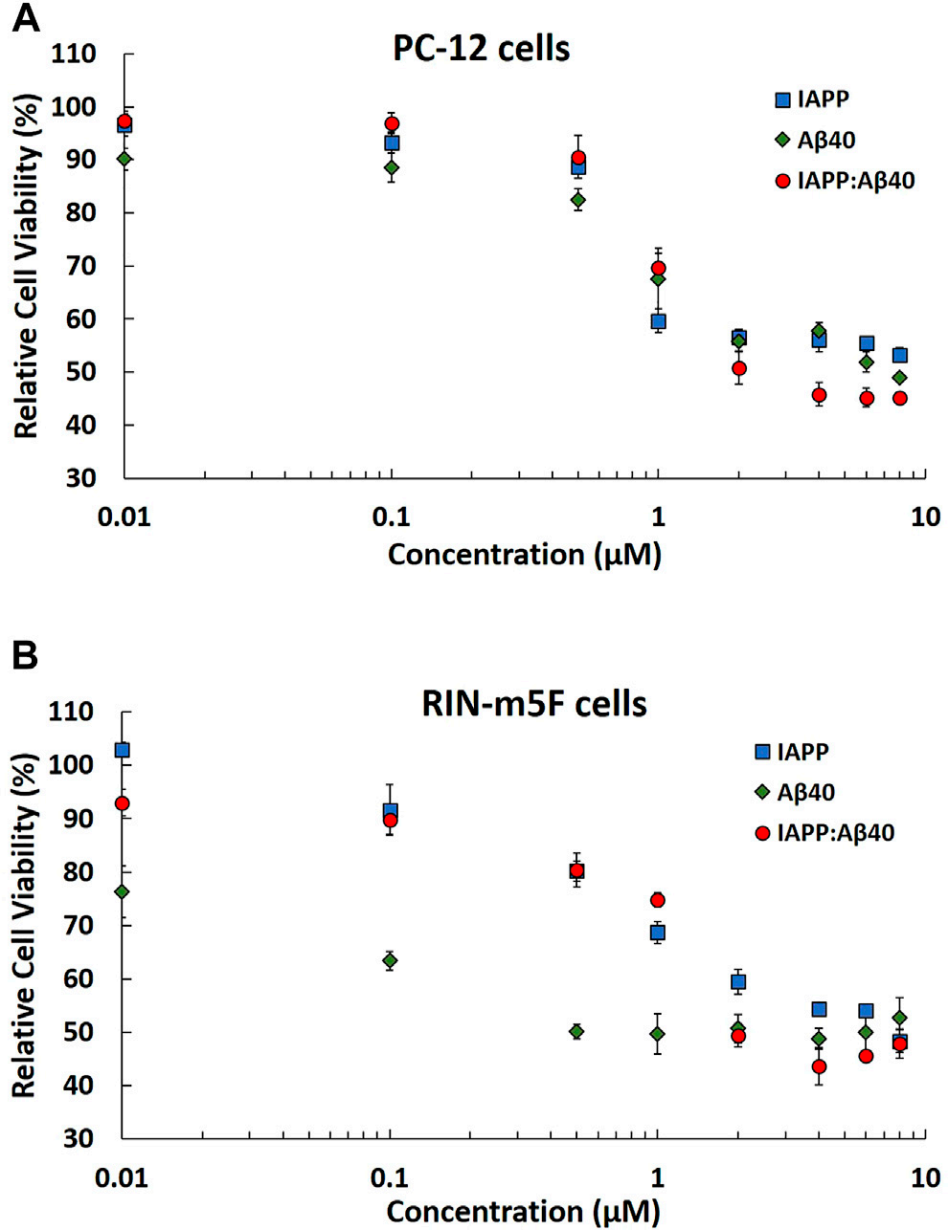
MTT results showing the normalized cell viability rates of **(A)** PC-12 cells and **(B)** RIN-m5F cells after treatment with 96 Hours-aged homo-aggregates (IAPP alone or Aβ40 alone) and hetero-aggregates (IAPP-Aβ40) at increasing concentrations (10 nM–8 µM). Normalized cell viability rates were calculated relative to control samples (cells without peptide treatment). Data represent mean ± SE (*n* = 3).

PC-12 and RIN-m5F cells were adopted as the neuronal cell model and pancreatic cell model for β-cells, respectively. The 96 h-aged homo-aggregates (IAPP alone or Aβ40 alone) and hetero-aggregates (equimolar mixture of IAPP-Aβ40) were assessed for their effect on the cell viabilities of PC-12 cells (Figure 4A) and RIN-m5F cells (Figure 4B) using the MTT assay. Samples of homo-aggregates and hetero-aggregates were diluted in serum-free cell culture media before addition to cells. Homo-aggregates (IAPP alone or Aβ40 alone) were added at final concentrations of 10 nM–8 µM. Hetero-aggregates (equimolar mixture of IAPP and Aβ40) were added at final concentrations of 10 nM–8 µM which represent the total concentration of both peptides (i.e., each peptide was present at 5 nM–4 µM).

Based on the MTT results in Figure 4A, IAPP and Aβ40 homo-aggregates are strongly toxic to PC-12 cells, specifically at concentrations ≥1 μM, which resulted in reducing the cell viability rates by at least 40 and 30% for IAPP alone and Aβ40 alone, respectively. Similarly, Figure 4B showed that IAPP and Aβ40 homo-aggregates were highly toxic to RIN-m5F cells resulting in reducing the cell viability rates by at least 32 and 50% for IAPP alone (≥1 µM) and Aβ40 alone (≥1 µM), respectively. However, in comparison to IAPP homo-aggregates, Aβ40 homo-aggregates showed higher toxicity towards RIN-m5F cells at low concentrations of 100 and 500 nM with viability rates <60%.

Next, we evaluated the concentration dependence toxicity of IAPP-Aβ40 hetero-aggregates on the two cell models to understand, at an *in vitro* level, the effect of co-aggregation on cell viabilities. Based on Figures 4A,B, IAPP-Aβ40 hetero-aggregates were consistently and almost equally toxic to both cell models with cell viability rates not exceeding 50% at concentrations ≥ 1 µM:1 µM for IAPP:Aβ40. For the lower concentration of hetero-aggregates, (IAPP:Aβ40, 0.5 µM:0.5 µM), PC-12 and RIN-m5F cells had cell viability rates of 69 and 74%, respectively, which indicate the toxic nature of the hetero-aggregates at these low concentrations. Although ThT results (Figures 1B,C) showed less enhancements in the ThT fluorescence for IAPP-Aβ40 mixture as compared to IAPP alone or Aβ40 alone, the cell viability data revealed that the hetero-aggregates are as toxic to both cell models as the homo-aggregates (see *Discussion* section).

In addition, given the potential cytotoxic effects of the oligomeric species, we tested the cytotoxicity of the oligomeric states of both individual (IAPP/Aβ40 alone) and mixed (IAPP-Aβ40) samples by adding the 3-Hours-aged homo-assemblies and hetero-assemblies to each cell model. As seen in Supplementary Figure S5, the MTT results revealed that the oligomeric states of IAPP-Aβ40 hetero-assemblies were highly toxic to both cell models (cell viability rates at 46 and 61% for PC-12 cells and RIN-m5F cells, respectively). In addition, the 3-Hours-aged IAPP homo-assemblies were toxic to both cell models whereas those of Aβ40 homo-assemblies were more toxic on RIN-5mF cells than on PC-12 cells. Taken together, MTT results (Figure 4 and Supplementary Figure S5) highlight the toxic effects of hetero-assemblies, at their oligomeric and fibrillar states, on both cell models.

## Results

### Evaluation of candidate inhibitors against Aβ40 and IAPP self-aggregation

Here, we investigated the inhibitory actions of 6 selected polyphenolic candidates in preventing Aβ40 and IAPP self-aggregation. The most potent candidate was further tested for its potency in preventing IAPP-Aβ40 co-aggregation as we present next.

**FIGURE 5:**
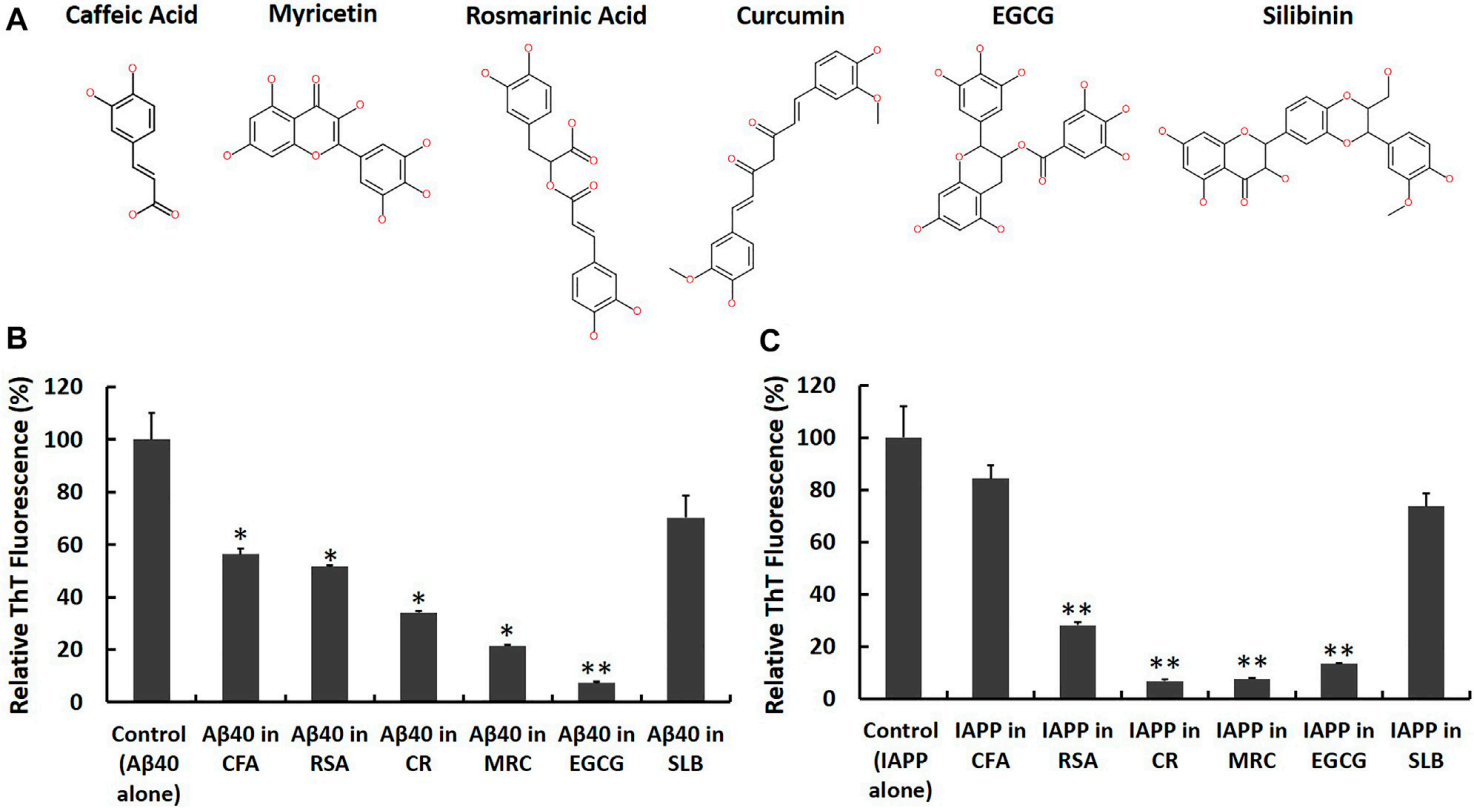
Screening candidate polyphenolic inhibitors against Aβ40 and IAPP self-aggregation using ThT fluorescence assay. **(A)** 2D chemical structures of the candidate inhibitors including CFA: Caffeic acid, RSA: Rosmarinic acid, CR: Curcumin, MRC: Myricetin, EGCG: (‐)-Epigallocatechin gallate and SLB: Silibinin. Samples containing **(B)** Aβ40 monomers (40 μM) and **(C)** IAPP monomers (40 µM) were prepared in absence and presence of an equimolar concentration of each candidate inhibitor and the ThT fluorescence intensities of aged samples were obtained and normalized relative to control samples, (fluorescence of sample/fluorescence of Aβ40 alone or IAPP alone) × 100. Data represent mean ± SE (*n* = 3). * *p*-value < 0.01, ** *p*-value < 0.001.

Aβ40 and IAPP are two highly amyloidogenic peptides that are intrinsically disordered and are marked with large interaction interfaces that initiate their self-interactions (Longhena et al., 2017). In order for inhibitors to prevent the aggregation of such peptides, they should theoretically block the sites of the highly plastic protein-protein interaction interfaces (Longhena et al., 2017). Despite these challenges, naturally-derived small molecules, specifically polyphenols, are commonly investigated for preventing Aβ40/IAPP self-aggregation (Porat et al., 2006; Dhouafli et al., 2018). In this research, we selected the polyphenols, Caffeic acid (Cheng et al., 2011; Chang et al., 2019), Myricetin (Ono et al., 2003, 2012; Shimmyo et al., 2008; DeToma et al., 2011; Zelus et al., 2012; Gargari et al., 2018; Dubey et al., 2021), Rosmarinic acid (Ono et al., 2012; Sun et al., 2019), Curcumin (Ono et al., 2004; Daval et al., 2010; Nedumpully-Govindan et al., 2016; Thapa et al., 2016), EGCG (Bastianetto et al., 2006; Ehrnhoefer et al., 2008; Bieschke et al., 2010; Meng et al., 2010; Liu et al., 2011; Cao and Raleigh, 2012; Suzuki et al., 2012; Cheng et al., 2013; Hyung et al., 2013; Zhang et al., 2013; Wang Q. et al., 2014; Xu et al., 2016) and Silibinin (Yin et al., 2011; Cheng B et al., 2012; Sciacca et al., 2017), as candidate inhibitors of Aβ40 and IAPP self-aggregation based on their known anti-aggregation activities in literature. Figure 5A demonstrates the chemical structures of the polyphenolic candidate inhibitors.

First, we co-incubated Aβ40 (40 µM) or IAPP (40 µM) with each candidate inhibitor at an equimolar concentration (i.e., 40 µM) and then measured the ThT fluorescence of aged samples in the absence and presence of each candidate inhibitor as shown in Figures 5B,C. The reduction rates of ThT fluorescence obtained from Figures 5B,C are shown in the second column of Table 1. Next, to examine whether the reported reductions in ThT fluorescence (Figures 5B,C) could quantify the actual inhibition of Aβ40 or IAPP self-aggregation, we conducted control experiments based on the study by (Hudson et al., 2009) as described in the Materials and Methods that test the possibility of the selected polyphenols in competitively binding with ThT on fibrils and/or in interfering with ThT fluorescence of preformed Aβ40 or IAPP fibrils. The control experiments (Supplementary Figures S6, S7) revealed that Curcumin dramatically reduced the ThT fluorescence of preformed Aβ40/IAPP fibrils which suggest that the reductions of ThT fluorescence in Figures 5B,C were highly biased by the quenching effect of Curcumin (40 µM) and hence may not indicate reduced fibril formation of either Aβ40 or IAPP. The control experiments showed some interference effects for each of Myricetin (40 µM), EGCG (40 µM) and Rosmarinic acid (40 µM) while Silibinin (40 µM) and Caffeic acid (40 µM) did not show any significant interference with the ThT fluorescence of preformed Aβ40/IAPP fibrils. The interference rate of each polyphenol with the ThT fluorescence spectra of preformed Aβ40/IAPP fibrils are presented in Table 1 (third column).

**TABLE 1:** Effective inhibition rates of Aβ40 (40 µM) or IAPP (40 µM) self-aggregation by each candidate inhibitor (40 µM) obtained by analyzing the results of the ThT screening experiments (Figure 5) and the reported ThT-interference rates (Supplementary Figures S6, S7).

Candidate polyphenols	Reduction rates of ThT fluorescence obtained by co-incubating each polyphenol with Aβ40/IAPP (Figure 5)^a^		Interference rate of each polyphenol with the ThT fluorescence of preformed Aβ40/IAPP fibrils (Supplementary Figures S6, S7)^b^		Effective inhibition of Aβ40/IAPP self-aggregation by each polyphenol^c^	
	Aβ40	IAPP	Aβ40	IAPP	Aβ40	IAPP
Caffeic Acid	43.7%	15.5%	-1.7%	-4.0%	45.4%	19.5%
Rosmarinic Acid	48.3%	71.7%	6.1%	32.0%	42.2%	39.7%
Curcumin	66.8%	93.1%	69.0%	90.8%	-2.2%	2.3%
Myricetin	78.0%	92.4%	34.0%	26.0%	44.0%	66.4%
EGCG	92.6%	86.5%	15.0%	19.0%	77.6%	67.5%
Silibinin	29.9%	26.3%	0.0%	1.3%	29.9%	25.0%

a Reduction rates of ThT fluorescence were obtained from Figure 5 by calculating the reductions of the normalized ThT fluorescence of Aβ40 or IAPP in the presence of each polyphenol relative to the control (Aβ40 alone/IAPP alone) × 100.

b Interference rates of ThT fluorescence were obtained from Supplementary Figures S6, S7 at λ_em_ 486 nm by calculating the reductions of the ThT fluorescence of preformed Aβ40/IAPP fibrils in the presence of each polyphenol relative to the control (preformed Aβ40/IAPP fibrils) × 100.

c Effective inhibition rates of self-aggregation were obtained by subtracting ThT interference rates^b^ from ThT reduction rates^a^.

Although the examined polyphenols were previously reported as candidate inhibitors of IAPP or Aβ40 self-aggregation, our work further investigated their inhibitory actions, under the same *in vitro* aggregation conditions, and examined the possibility of their interference with ThT fluorescence of preformed fibrils. As demonstrated in Table 1 (fourth column), the effective inhibition rate of Aβ40 or IAPP self-aggregation by each candidate inhibitor was obtained by combining the results of the ThT screening experiments (Figure 5) and the reported ThT fluorescence interference rates (Supplementary Figures S6, S7).

Table 1 shows that EGCG exhibited the highest rates of inhibition against Aβ40 and IAPP self-aggregation by 67.5 and 77.6%, respectively. In addition to EGCG, Myricetin, and Rosmarinic acid were also shown to have potential inhibitory roles against both Aβ40 and IAPP self-aggregation but with rates that are lower than those observed for EGCG. Next, Caffeic acid and Silibinin did not have high inhibition rates against both peptides. For Silibinin examined in our experiments, it consisted of two diastereomers (silybin A and silybin B). Hence, the prevention rates of Silibinin presented in Table 1 refer to the effect of both diastereomers. However, a study by Sciacca et al., which investigated the inhibitory roles of four optically pure components of Silymarin, demonstrated that only silybin B had the highest inhibition potency among the examined Silymarin components (Sciacca et al., 2017). Hence, the results of the previous study (Sciacca et al., 2017) can explain the partial inhibition rates observed for the Silibinin that we used in our screening experiments.

Among the tested polyphenols in our experiments, EGCG demonstrated the highest inhibition action against Aβ40 and IAPP self-aggregation. Hence, we selected EGCG as the most potent model candidate to investigate its inhibitory action against IAPP-Aβ40 co-aggregation as will be presented next. To further support the ThT results, STEM images were acquired for aged samples of Aβ40 alone and Aβ40-EGCG as well as IAPP alone and IAPP-EGCG (Supplementary Figure S8). Less fibrils were depicted for IAPP-EGCG whereas non-fibrillar aggregates were observed for Aβ40-EGCG.

### Effect of EGCG on IAPP-Aβ40 Co-Aggregation Kinetic Profiles

**FIGURE 6:**
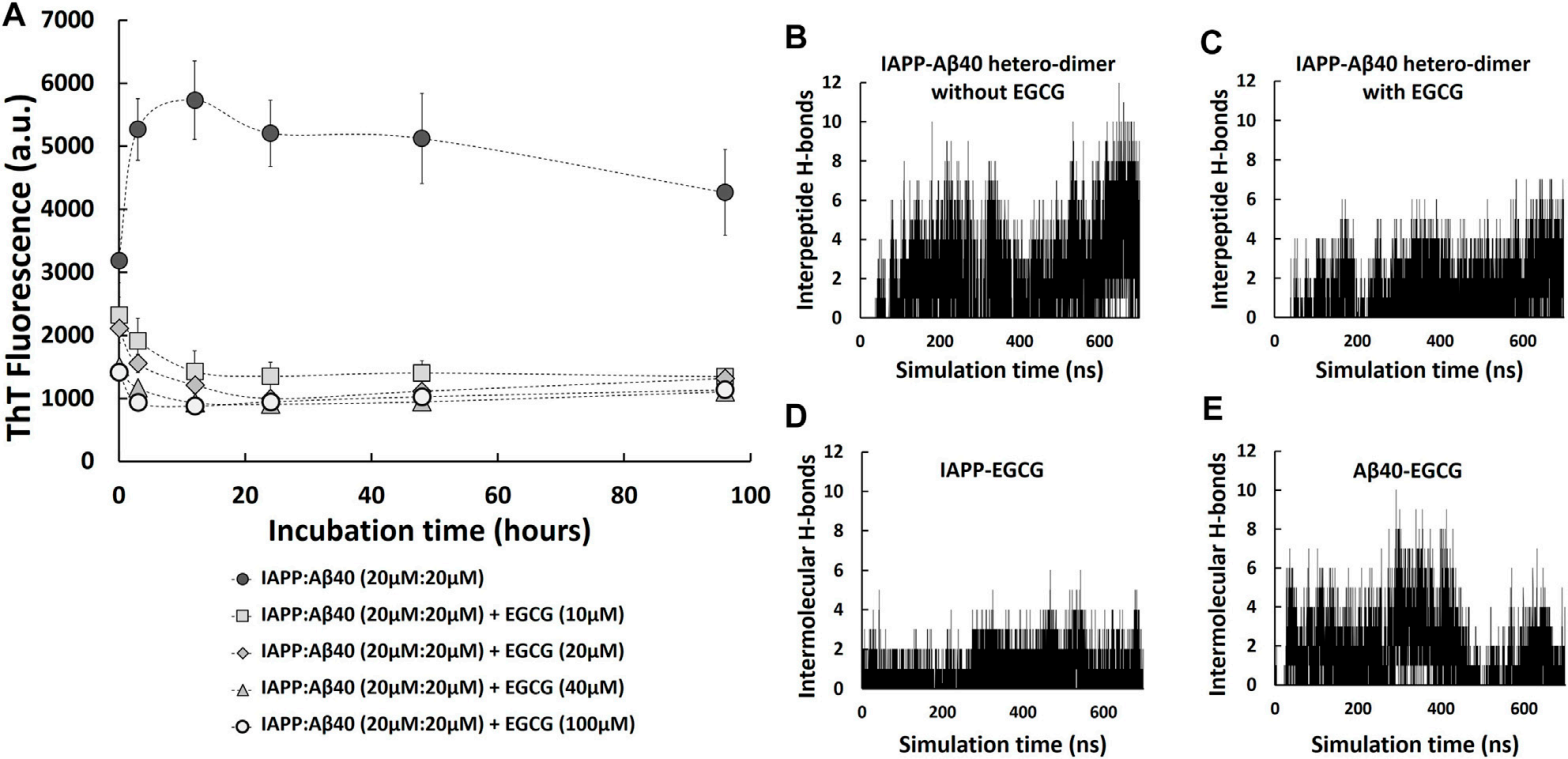
IAPP-Aβ40 co-aggregation kinetic profiles in the absence and presence of EGCG. **(A)** IAPP-Aβ40 (20 µM:20 µM) samples were co-incubated with increasing EGCG concentrations (10, 20, 40, and 100 µM) and at the indicated time-points, the ThT fluorescence measurements were acquired. Data represent mean ± SE (*n* = 3). Time evolution of inter-peptide hydrogen bonds formed between IAPP and Aβ40 in the absence **(B)** and presence **(C)** of five EGCG molecules. Time evolution of inter-molecular hydrogen bonds formed between the five EGCG molecules and each of IAPP **(D)** and Aβ40 **(E)** in the hetero-dimer system.

Given the potential pathological effect of the detected amyloid co-deposition of IAPP-Aβ40 in brain and pancreatic tissues, we believe that attempting to prevent the amyloid cross-interaction would be significant in addressing the associated link between AD and T2D at the protein level. Specifically, we examined how EGCG affects the kinetic pathway of the co-aggregation process, how it changes the secondary structure and morphologies of the hetero-aggregates and to what extent it could minimize the induced cytotoxicity of the hetero-aggregates. To test the effect of EGCG in inhibiting IAPP-Aβ40 co-aggregation, we first co-incubated equimolar mixture of IAPP:Aβ40 (20 µM:20 µM) in the presence of increasing EGCG concentrations (10 μM, 20 μM, 40 μM and 100 µM) and the ThT fluorescence was measured for all samples at selected time-points for a duration of 96 h as shown in Figure 6A. In the prepared samples, EGCG concentration was either lower than (0.25-fold and 0.5-fold), equivalent to, or higher than (2.5-fold) the total concentration of IAPP and Aβ40. Over the course of the experiment, all the examined EGCG concentrations showed significant reductions in ThT fluorescence values in comparison to IAPP-Aβ40 samples in the absence of EGCG (Figure 6A). Dose-dependent reductions in ThT fluorescence of the mixed sample in the presence of EGCG were detected at the first three time points of the ThT assay after which the reduction rates of ThT in the presence of all EGCG concentrations converged to the same levels. The reduction rates of ThT fluorescence obtained from Figure 6A at the 96-Hours time-point are shown in the second column in Table 2.

**TABLE 2:** Effective inhibition rates of IAPP-Aβ40 (20 µM:20 µM) co-aggregation by EGCG (10–100 µM) obtained by analyzing the results of the ThT fluorescence reduction rates (Figure 6A) and ThT fluorescence interference rates (Supplementary Figure S9).

EGCG concentration	Reduction rates of ThT fluorescence obtained by co-incubating IAPP-Aβ40 with EGCG (Figure 6A)^a^	EGCG interference effect with ThT fluorescence of preformed IAPP-Aβ40 hetero-aggregates (Supplementary Figure S9)^b^	Effective inhibition of IAPP-Aβ40 co-aggregation by EGCG^c^
10 µM	72.4%	8.9%	63.5%
20 µM	72.8%	16.6%	56.2%
40 µM	77.2%	23.6%	53.6%
100 µM	76.6%	35.6%	41.0%

a Reduction rates of ThT fluorescence were obtained from Figure 6A by calculating the reductions of the ThT fluorescence intensities of IAPP-Aβ40-EGCG relative to the control (IAPP-Aβ40 alone) at the 96-Hour time-point.

b Interference rates of ThT fluorescence were obtained from Supplementary Figure S9 at λ_em_ 486 nm by calculating the reductions of the ThT fluorescence of preformed IAPP-Aβ40 hetero-aggregates in the presence of EGCG relative to the control (preformed IAPP-Aβ40 hetero-aggregates) ×100.

c Effective inhibition rates of hetero-aggregate were obtained by subtracting ThT interference rates^b^ from ThT reduction rates^a^.

As we demonstrated earlier, EGCG had an interference effect when added to the mixture of preformed Aβ40/IAPP fibrils and ThT as it reduced the ThT fluorescence of preformed Aβ40/IAPP fibrils at rates of 15 and 19%, respectively (third column of Table 1, Supplementary Figures S6, S7). Hence, we also tested for the interference effect of EGCG (10–100 µM) with the ThT fluorescence of preformed IAPP-Aβ40 hetero-aggregates (96-Hours aged) as shown in Supplementary Figure S9. The interference rates of EGCG (10–100 µM) with the ThT fluorescence of preformed IAPP-Aβ40 hetero-aggregates are presented in Table 2 (third column) where a dose dependent interference effect was observed ranging between 9 and 36% for 10–100 µM of EGCG. As demonstrated in Table 2, the effective inhibition rates of IAPP-Aβ40 co-aggregation by EGCG were obtained by combining the results of the ThT fluorescence reduction rates (Figure 6A) and ThT fluorescence interference rates (Supplementary Figure S9). The effective inhibition rates of EGCG against IAPP-Aβ40 co-aggregation were found to range between 41 and 63.5%, suggesting the potential inhibitory roles of EGCG against hetero-aggregate formation despite its interference effects with the ThT dye.

Next, to examine role of EGCG in modulating IAPP-Aβ40 cross-interaction, we used MD simulations to study the formation of IAPP-Aβ40 hetero-dimer in the presence of EGCG. Figures 6B,C show the time evolution (700 ns) of inter-peptide hydrogen bonds at the dimer interface between IAPP and Aβ40 in the absence and presence of EGCG, respectively. The hetero-dimer formation in the presence of EGCG had an overall smaller number of hydrogen bonds between IAPP and Aβ40 as compared to that formed in the absence of EGCG. The low number of inter-peptide hydrogen bonds between IAPP and Aβ40 in the presence of EGCG is likely due to the inter-molecular hydrogen bonds that form between the five EGCG molecules and each of IAPP and Aβ40 as demonstrated in Figures 6D,E. The five EGCG molecules form more intermolecular hydrogen bonds with Aβ40 than with IAPP throughout the simulation time. Overall, EGCG interactions with both peptides interfere with the formation of a stable hetero-dimer interface which suggest that EGCG can have an early inhibitory role against IAPP-Aβ40 cross-interaction and co-aggregation (see Supplementary Figure S1B) for a representative snapshot of IAPP-Aβ40-EGCG complex.

### Effect of EGCG on the Morphology of IAPP-Aβ40 Hetero-Aggregates

**FIGURE 7:**
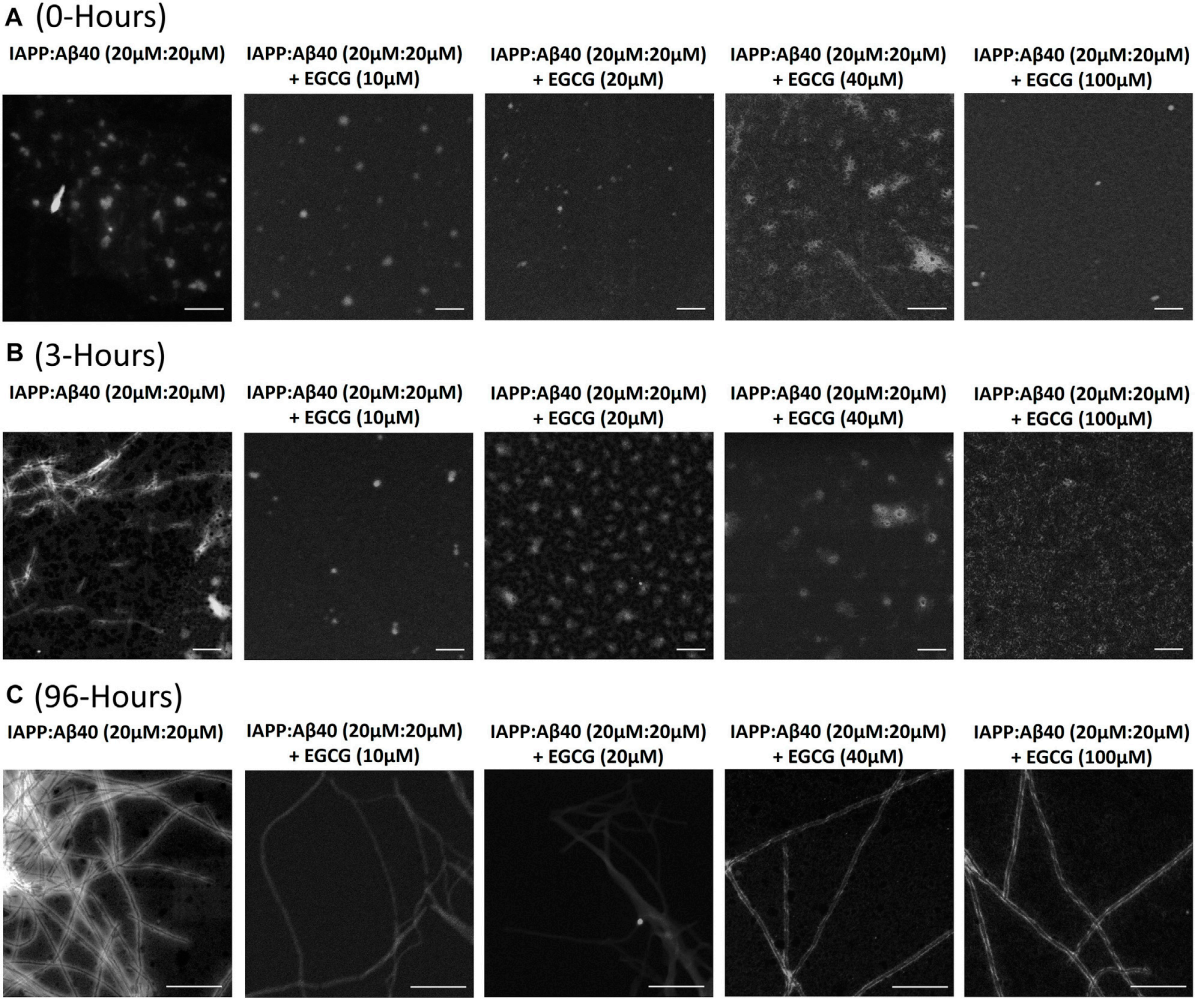
Morphological characterization using scanning transmission electron microscopy (STEM) of **(A)** 0-Hour, **(B)** 3-Hours and **(C)** 96-Hours assemblies formed by the co-aggregation of equimolar IAPP-Aβ40 (20 µM: 20 µM) in the absence and presence of increasing EGCG concentrations (10–100 µM). All scale bars represent 200 nm.

Figure 7 and Supplementary Table S3 demonstrate the STEM images and dimensions of the assemblies formed by the co-aggregation of IAPP-Aβ40 (20 µM:20 µM) in the absence and presence of increasing EGCG concentrations (10, 20, 40, and 100 µM). All samples were imaged at three time-points during the co-aggregation process, the initial time-point (0-Hours, Figure 7A), an early time-point (3-Hours, Figures 7B) and a late time-point (96-Hours, Figure 7C).

At the first time-point (0-Hour), IAPP-Aβ40 formed assemblies with an average diameter of 38.4 ± 5.4 nm. In the presence of EGCG, IAPP-Aβ40-EGCG complexes formed assemblies that are on average similar but slightly smaller than those without EGCG (Supplementary Table S3).

At 3-Hours of incubation, IAPP-Aβ40 hetero-assemblies were mainly populated by short fibrils (fibril diameter 12.0 ± 2.2 nm) in addition to other amorphous aggregates (Figure 7B and Supplementary Figure S3). In contrast, the addition of EGCG, at all concentrations, prevented the early fibrillar formation where non-fibrillar structures were only observed. Preventing the early fibrillar formation can be one of the mechanisms by which EGCG interferes with IAPP-Aβ40 co-aggregation. In specific, IAPP-Aβ40 samples containing EGCG at 100 µM had relatively smaller sizes of amorphous aggregates (diameter 16.2 ± 4.7 nm) in comparison to those observed for EGCG at 40 µM (diameter 44.0 ± 5.2 nm), 20 µM (diameter 62.1 ± 10.6 nm) or 10 µM (diameter 39.8 ± 9.3 nm). We performed MTT tests and found that the non-fibrillar assemblies of IAPP-Aβ40 in the presence of EGCG have reduced toxicity as compared to those formed without EGCG (see Supplementary Figure S10).

At the last time-point of the co-aggregation process (96-Hours), IAPP-Aβ40 samples formed condensed network of mature fibrils with a fibril diameter of 9.5 ± 1.7 nm (Figure 7C). The acquired STEM images of the mixed samples incubated with different EGCG concentrations revealed fibril presence albeit with less amount than the IAPP-Aβ40 alone. The fibrils formed in the presence of EGCG were morphologically similar to those formed without EGCG and had slightly larger fibril diameters (Supplementary Table S3).

### Effect of EGCG on the Secondary Structure of IAPP-Aβ40 Hetero-Aggregates

**FIGURE 8:**
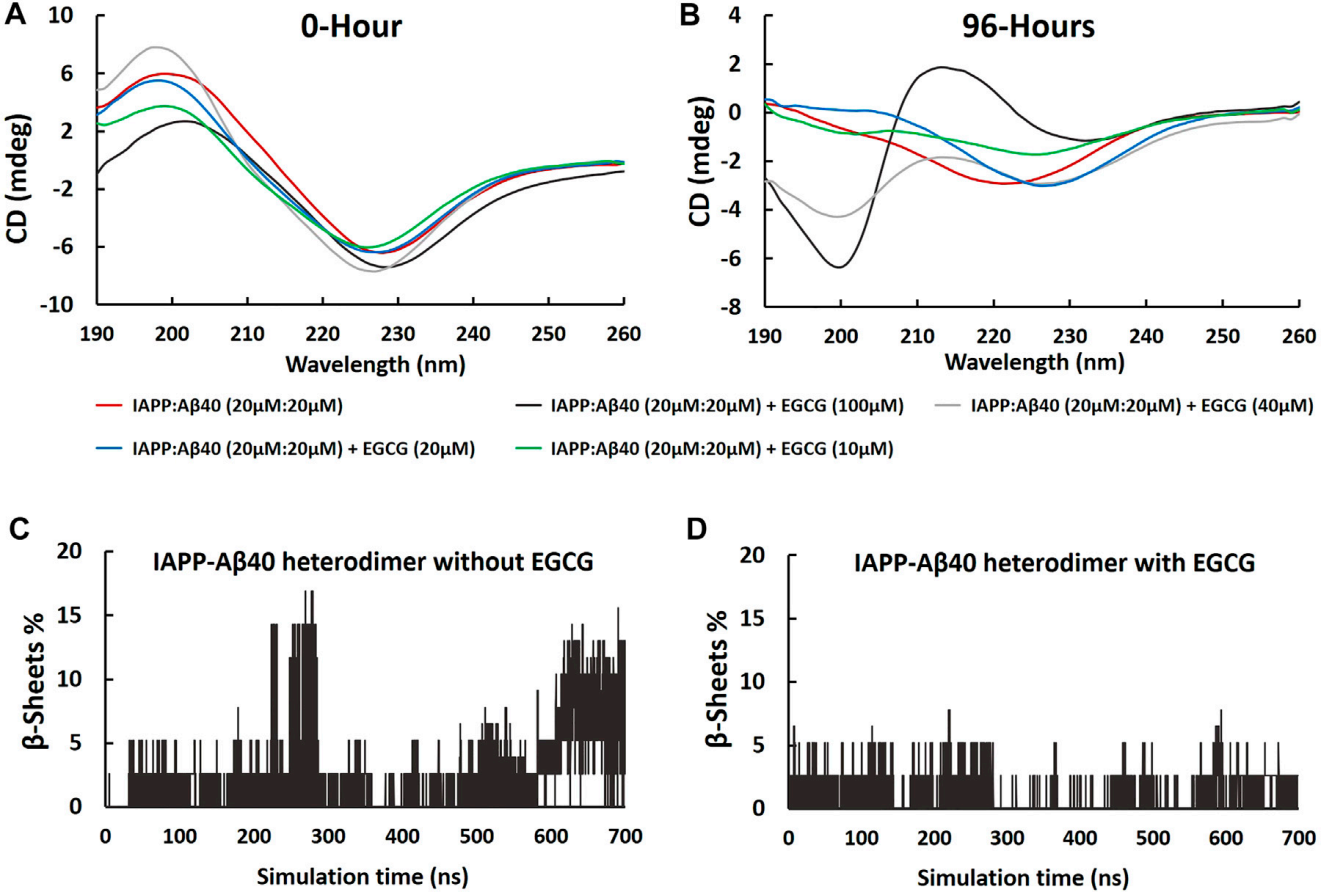
Far-UV CD spectra of samples containing equimolar IAPP-Aβ40 (20 µM:20 µM) in the absence and presence of increasing EGCG concentrations (10–100 µM) at **(A)** 0-Hour (fresh preparations) and **(B)** 96-Hours (aged aggregates). Percentages of residues forming β-sheets (β-strands and β-bridges) in IAPP-Aβ40 hetero-dimer, in the absence **(C)** and presence of EGCG **(D)**, were obtained using the 700 ns MD trajectories.

The CD spectra of the fresh and aged IAPP-Aβ40 (20 µM:20 µM) samples in the absence and presence of increasing EGCG concentrations (10–100 µM) are presented in Figure 8 and the deconvolution data of CD spectra are shown in Supplementary Table S4. Although the presence of EGCG did not result in major peak shifts of IAPP-Aβ40 spectrum measured at 0-Hour (Figure 8A), however, EGCG slightly modulated the secondary structural elements (Supplementary Table S4). Specifically, reductions in the content of β-sheets, and increases in the unordered structures or alpha-helices of IAPP-Aβ40 were observed with the different concentrations of EGCG. Specifically, EGCG at 100 μM, had the highest increase in the unordered structures by 8%. In addition, we analyzed the secondary structure of each residue of IAPP-Aβ40 hetero-dimer (in the absence and presence of EGCG) using MD trajectories (700 ns). As seen in Figures 8C,D, the content of β-sheets, consisting of β-strands and β-bridges, was higher the in the hetero-dimer formed without EGCG indicating that EGCG interaction with each of Aβ40 and IAPP reduces the formation of β-sheets in both IAPP and Aβ40 residues.

Figure 8B shows the CD spectrum of the 96 Hours-aged IAPP-Aβ40 hetero-aggregates which had a single negative peak at around 220 nm reflecting the dominance of β-sheet-rich fibrils (53.3% of β-sheets as estimated by deconvolution). By visual inspection, the presence of EGCG led to major peak changes in the CD spectrum of 96 Hours-aged IAPP-Aβ40 (Figure 8B). In particular, the spectra of the IAPP-Aβ40 in the presence of EGCG at 40 and 100 µM had new negative peaks at the early wavelengths (around 200 nm) and at the higher wavelengths (227 and 233 nm); suggesting the formation of more unordered structures and less β-sheets in the hetero-aggregate end products. Interestingly, the spectrum of IAPP-Aβ40 with EGCG (100 µM) had a broad positive peak at 212 nm, which also suggest the presence of unordered structures. Based on Supplementary Table S4, the presence of EGCG at 100 and 40 µM led to major changes in the secondary structural elements of the IAPP-Aβ40 spectrum as seen in the increases of unordered structures by 14 and 9% (respectively), the noticeable decreases of β-sheets by 19% and the increases in alpha helices by 4.4 and 8.4% (respectively). At lower concentrations of EGCG, the CD spectra experienced a peak shift towards the higher wavelengths, approximately 226 nm, in the presence of EGCG at 10 and 20 μM, which is estimated by the deconvoluted data to reduce the β-sheet contents of the mixed sample by 5 and 8%, respectively.

### Effect of EGCG on the Cytotoxicity of IAPP-Aβ40 Hetero-Aggregates

Given that EGCG is an antioxidant polyphenol, we first tested whether it interferes with MTT by measuring the absorbance at 570 nm of samples containing increasing EGCG concentration (0.5–40 µM) with MTT only (without cells) as shown in Supplementary Figure S11. Based on the results in Supplementary Figure S11, we selected EGCG concentrations ≤10 µM to be tested for their effect in preventing the cytotoxicity of IAPP-Aβ40 hetero-aggregates as these EGCG concentrations were shown to minimally interfere with MTT absorbance. Additionally, we tested the effect of EGCG alone (0.5–40 µM) on each cell model (Supplementary Figure S12) to examine whether EGCG could have its own effects on the cell viabilities and the results showed no detectable effects of EGCG alone on the cell viabilities of PC-12 cells or RIN-m5F cells.

**FIGURE 9:**
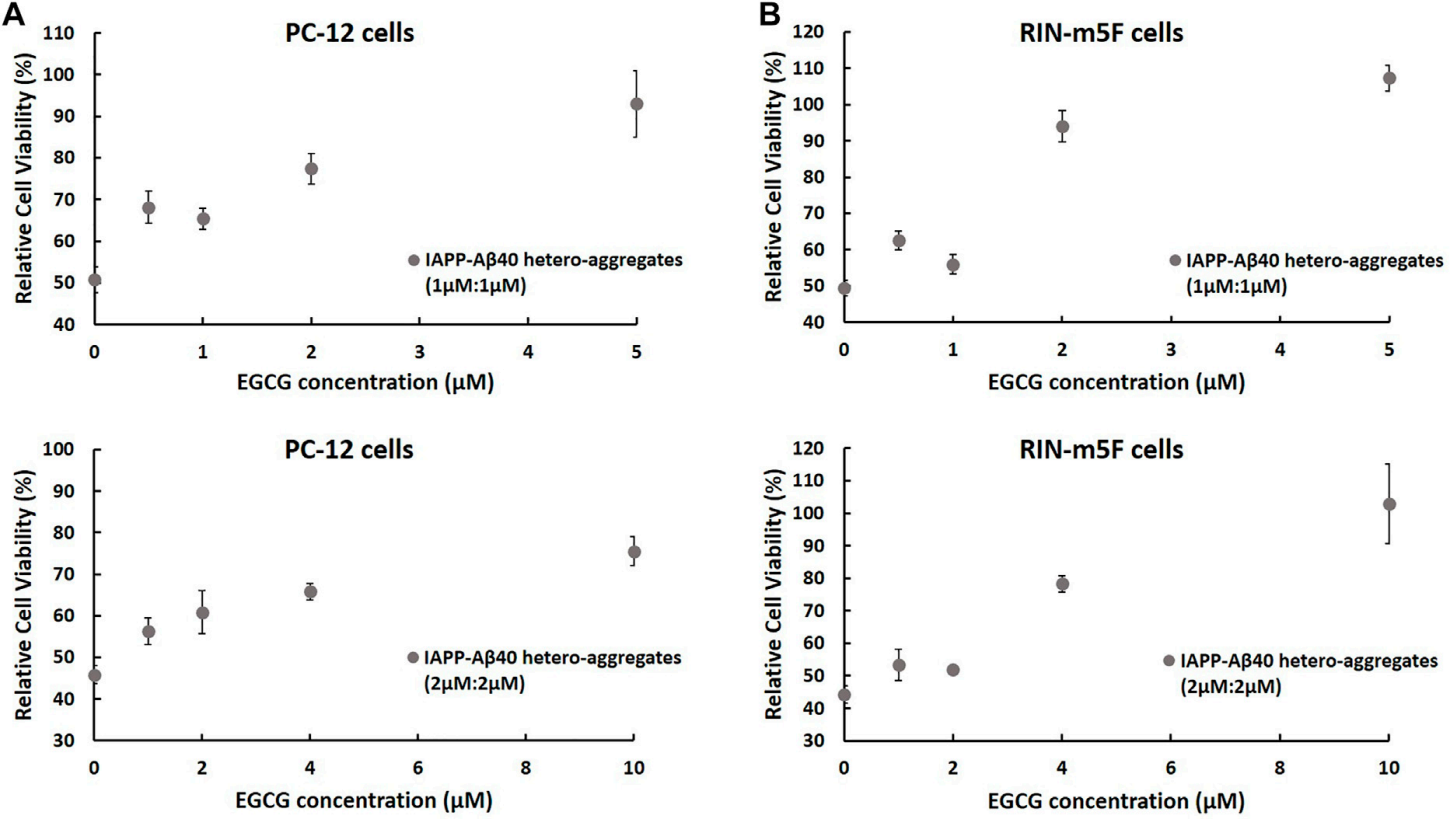
MTT results showing the normalized cell viability rates of **(A)** PC-12 cells and **(B)** RIN-m5F cells after treatment with 96 Hours-aged IAPP-Aβ40 hetero-aggregates at two concentrations (1 µM:1 µM; upper panels) and (2 µM:2 µM; lower panels) in the absence and presence of increasing EGCG concentrations. EGCG concentrations were either lower than (0.25-fold and 0.5-fold), equivalent to, or higher than (2.5-fold) the total concentration of IAPP and Aβ40. Normalized cell viability rates were calculated relative to control samples (cells without the peptide or inhibitor treatment). Negative control or the background values of the interference of EGCG with MTT only (no cells) were obtained from Supplementary Figure S11, and subtracted from the absorbance values of cells treated with matching EGCG concentrations. Data represent mean ± SE (*n* = 3), * *p*-value < 0.001.

Next, given that IAPP-Aβ40 hetero-aggregates showed consistent toxic effects (Figure 4), we examined if EGCG could minimize the toxicity of IAPP-Aβ40 hetero-aggregates. In specific, we used MTT to examine the protective effect of increasing EGCG concentrations against two concentrations of the 96-Hours-aged hetero-aggregates, 1 µM:1 µM (upper panels of Figure 9, 2µM:2 µM (lower panels of Figure 9). Similar to the ThT experiments, we tested EGCG concentrations that were lower than (0.25-fold and 0.5-fold), equivalent to, or higher than (2.5-fold) the total concentration of IAPP and Aβ40.

MTT results in Figure 9 show increased cell viability rates of both cell lines after treatment with IAPP-Aβ40 hetero-aggregates that were prepared in the presence of EGCG. The protective effect of EGCG was dose-dependent with the highest EGCG concentrations, which are 2.5-fold higher than the total concentration of IAPP and Aβ40, exerting strong protective actions towards both cell lines. Particularly, IAPP-Aβ40 (1 µM:1 µM) in the presence of 5 µM EGCG had enhancements in cell viability rates by 42 and 58% for PC-12 cells and RIN-m5F cells, respectively. Similarly, IAPP-Aβ40 (2 µM:2 µM) in the presence of 10 µM EGCG had increased viability rates by 30 and 60% for PC-12 cells and RIN-m5F cells, respectively. The lower EGCG concentrations, which are equivalent to the total concentration of IAPP and Aβ40, had slightly less protective effect as the enhancements in cell viability rates measured between 20 and 44% for both cell models. However, EGCG concentrations that are lower than (0.25-fold and 0.5-fold) the total concentration of IAPP and Aβ40 had minimally increased the cell viability rates of both cell lines, with enhancement rates ranging between 7–17%.

To support MTT results, the cytotoxic effects of IAPP-Aβ40 hetero-aggregates in the absence and presence of EGCG were assessed using Live/Dead assay. As shown in Supplementary Figures S13, S14, the cell viability rates of PC-12 cells and RIN-m5F cells were reduced when treated with IAPP-Aβ40 hetero-aggregates (without EGCG addition), whereas cells treated with IAPP-Aβ40 hetero-aggregates in the presence of EGCG had higher viability rates indicating the inhibitory role of EGCG against the cytotoxicity of IAPP-Aβ40 hetero-aggregates.

## Discussion

The self-aggregation of the two amyloidogenic peptides, Aβ and IAPP, is implicated in the pathology of AD and T2D, respectively (Murphy and LeVine, 2010; Jurgens et al., 2011; Chiti and Dobson, 2017). However, recent findings highlight the role of Aβ and IAPP cross-interaction in mediating the cross-talk of AD and T2D at a protein level (Morales et al., 2009, 2013; Miklossy et al., 2010; Jackson et al., 2013; Martinez-Valbuena et al., 2019, 2021; Raimundo et al., 2020; Stanciu et al., 2020; Zhang et al., 2021). Despite the potential pathological effect of IAPP-Aβ40 co-deposition in brain and pancreatic tissues (Miklossy et al., 2010; Jackson et al., 2013; Oskarsson et al., 2015; Martinez-Valbuena et al., 2019, 2021), previous literature has mainly focused on preventing the self-aggregation but not the co-aggregation pathways of IAPP and Aβ40. Given that co-aggregation mechanisms can result in cytotoxic hetero-aggregates, our work investigated the prevention of IAPP and Aβ40 co-aggregation using a model polyphenolic inhibitor.

In particular, we first characterized IAPP-Aβ40 hetero-aggregates by examining their kinetic co-aggregation pathways, secondary structure, morphological changes and cytotoxic effects on neuronal and pancreatic cell models. Our results indicated that monomeric IAPP and Aβ strongly co-interact to form hetero-assemblies that undergo a distinct co-aggregation pathway resulting in β-sheets-rich fibrils which, despite being less in amount than those formed by the self-aggregation of each peptide, have high cytotoxic rates on both PC-12 and RIN-m5F cells.

Among previous studies that examined the *in vitro* co-aggregation of IAPP and Aβ40/42 (Yan et al., 2007, 2014; Hu et al., 2015; Young et al., 2015; Ge et al., 2018; Bharadwaj et al., 2020), only few investigated the cellular toxicity of hetero-aggregates in comparison to homo-aggregates (Yan et al., 2007, 2014; Bharadwaj et al., 2020). In two of these studies (Yan et al., 2007, 2014), the authors reported that the cross-interaction of monomeric and pre-fibrillar IAPP and Aβ40 delays their fibrillogenesis but the aged (7 days) hetero-aggregates were as toxic as the homo-aggregates. Another recent study demonstrated that IAPP-Aβ42 hetero-aggregates have an exacerbated neurotoxicity when compared to IAPP alone or Aβ42 alone (Bharadwaj et al., 2020). Hence, our data (Figure 4) further support the findings of previous studies as we demonstrated the consistent toxic effect of the hetero-aggregates formed by the cross-interaction of IAPP and Aβ40 on a neuronal cell model and a pancreatic cell model for β-cells.

The *in vitro* cytotoxic results of IAPP-Aβ40 hetero-aggregates presented in our work and in previous studies (Yan et al., 2007, 2014, 201; Bharadwaj et al., 2020) suggest an *in vivo* pathological roles of IAPP-Aβ co-deposits which were found to be populated in the brain (Jackson et al., 2013; Oskarsson et al., 2015; Martinez-Valbuena et al., 2019, 2021) and the pancreatic tissues of patients (Miklossy et al., 2010; Martinez-Valbuena et al., 2019, 2021). One of these studies (Jackson et al., 2013) showed that the brain tissues infiltrated by IAPP and IAPP-Aβ deposits were morphologically different than those of the control group where capillary bending, cell multi-nucleation and cell variation in nuclear sizes were observed in the affected tissues (Jackson et al., 2013). In addition, the reported accumulation of IAPP oligomers in the cerebrovasculature and brain gray matter may lead to pathological effects including prevention of Aβ clearance which contribute to AD pathology (Jackson et al., 2013). Similarly, the accumulation of Aβ and Tau deposits in the pancreatic β-cells of AD and neurological asymptomatic T2D patients can further impair insulin resistance and contribute to T2D pathology (Martinez-Valbuena et al., 2019). Although IAPP can enter brain from circulation (Banks et al., 1995; Banks and Kastin, 1998), it was shown that IAPP can be expressed in the brain (Mulder et al., 1995; Fawver et al., 2014). In fact, the detected IAPP levels in both plasma (Percy et al., 1996) and brain (Fawver et al., 2014) is in the pico-molar range. Similarly, Aβ levels are in the pico-molar to nano-molar range (Qiu et al., 2015; Novo et al., 2018). In fact, Aβ40 and Aβ42 are the two most prominent isoforms of the β-amyloid peptides (Qiu et al., 2015). Although Aβ42 is more amyloidogenic and toxic with respect to Aβ40, the higher abundance of Aβ40 in body fluids, with a ratio of 9:1 (Aβ40:Aβ42) (Sciacca et al., 2017), is the reason for adopting Aβ40 in our *in vitro* co-aggregation experiments.

In summary, the concentration dependence toxicity data (Figure 4) presented in our work, although at concentrations higher than the *in vivo* physiological ones, provide an insight into the toxic effect of the hetero-aggregates formed by the cross-interaction of IAPP and Aβ. Despite the pathological effects of both self- and co-deposits, previous literature has mainly focused on preventing the self-aggregation but not the co-aggregation pathways of IAPP and Aβ. Hence, our study examined the use of a small molecule inhibitor, EGCG, for targeting the co-aggregation of IAPP and Aβ40. It is important to note that the proposed co-aggregation inhibition mechanism can be implemented in addition to the well-studied self-aggregation inhibition as both approaches constitute promising strategies in devising preventative therapies for the reported clinical association between Alzheimer’s disease and Type 2 diabetes.

In fact, EGCG is among the well-investigated polyphenols for the prevention of self-aggregation where several previous reports have pointed towards its various mechanisms to interfere with different phases of IAPP or Aβ self-aggregation processes (Bastianetto et al., 2006; Ehrnhoefer et al., 2008; Bieschke et al., 2010; Meng et al., 2010; Liu et al., 2011; Cao and Raleigh, 2012; Suzuki et al., 2012; Cheng et al., 2013; Hyung et al., 2013; Zhang et al., 2013; Wang Q. et al., 2014; Xu et al., 2016). An early study showed that EGCG was the most potent flavan-3-ol of the black and green tea extracts in preventing Aβ42 oligomerization and fibrillation and thus protecting rat hippocampal cells against Aβ42-induced cytotoxicity (Bastianetto et al., 2006). Similarly, EGCG efficiently inhibited IAPP fibril formation and protected the rat β-cells from the toxic effect of IAPP fibrils (Meng et al., 2010). A study showed that the gallate ester of EGCG and the terminal hydroxyl groups of its tri-hydroxyl-phenyl ring are critical structural elements for its inhibitory actions (Cao and Raleigh, 2012).

In our study, we extended the use of EGCG by demonstrating its inhibitory functions against IAPP-Aβ40 co-aggregation using experimental and computational approaches. Although the ThT assay demonstrated a wide range of effective EGCG concentrations against IAPP-Aβ40 co-aggregation (Figure 6A), the results of CD (Figure 8) and MTT (Figure 9) enabled us to further determine the most effective inhibitory concentrations of EGCG. The results demonstrate that the higher concentrations of EGCG, which are either equivalent to or are 2.5-fold higher than the total concentration of IAPP and Aβ40, are more effective in interfering with the cross-interaction of IAPP-Aβ40 as seen in the kinetic profiles (Figure 6A), MD results (Figure 6B–E), STEM images (Figure 7) and CD spectral changes (Figure 8). Importantly, these two concentrations were most effective in protecting PC-12 and RIN-m5F cells against the IAPP-Aβ40 hetero-aggregates toxicity than the lower EGCG concentrations (Figure 9). The main aim of our work is to demonstrate the ability of small molecules to inhibit IAPP-Aβ40 hetero-aggregation. For this, we used EGCG as a model inhibitor. However, future studies shall address a detailed evaluation of the inhibitory potentials of different polyphenols against hetero-aggregation of IAPP-Aβ40 and against hetero-aggregation of other pathogenic proteins.

The *in vitro* co-aggregation data presented here and in previous studies (Yan et al., 2007, 2014; Hu et al., 2015; Young et al., 2015; Ge et al., 2018; Bharadwaj et al., 2020), emphasize the significance of the cross-amyloid mechanism in establishing the association between AD and T2D. In addition to IAPP and Aβ40 co-aggregation, the co-aggregation of other proteins such as Tau and α-Synuclein (Giasson, 2003), IAPP and α-Synuclein (Horvath and Wittung-Stafshede, 2016), IAPP and Tau-Fragment R3 (Arya et al., 2019), Aβ and α-Synuclein (Luo et al., 2016; Bhasne and Mukhopadhyay, 2018; Köppen et al., 2020) were reported which can elucidate the cross-talk of other protein misfolding diseases (Luo et al., 2016; Bharadwaj et al., 2017; Ren et al., 2019; Konstantoulea et al., 2021a). Given that cross-interaction can result in unique assemblies and cytotoxic hetero-aggregates, it is important to investigate the prevention of such processes which is also a strategy suggested by a review addressing the cross-interaction of Aβ with 10 amyloid-related proteins (Luo et al., 2016). Our current results can be further tested against the co-aggregation of other amyloidogenic peptides both *in vitro* and *in vivo* in an attempt to address the cross-talk of different protein misfolding diseases beyond AD and T2D.

## Conclusion

The self-aggregation of the two amyloidogenic peptides, Aβ40 and IAPP, is implicated in the pathology of AD and T2D, respectively. However, recent findings highlight the role of Aβ40 and IAPP cross-interaction in mediating the cross-talk of AD and T2D at a protein level. Despite the pathological effects of both self- and co-deposits, previous literature has mainly focused on interfering or preventing the self-aggregation but not the co-aggregation pathways of IAPP and Aβ40. In this research, we investigated the use of a small molecule, EGCG, in inhibiting the co-aggregation of IAPP-Aβ *in vitro*. Experimental and computational characterizations of IAPP-Aβ40 revealed that monomer mixing of the two peptides results in the formation of hetero-dimers (Figure 1E) that are stabilized with inter-peptide H-bonds, as well as the formation of hetero-complexes (Figures 2, 3) that undergo distinct co-aggregation pathways (Figures 1B,C) which yield less amounts of hetero-aggregate as compared with homo-aggregates. The hetero-aggregates were shown to consistently exert toxic effects similar to those shown by homo-aggregates (Figure 4). Hence, we believe that interfering with the formation of hetero-aggregates is as significant as interfering with homo-aggregates. We next examined the inhibitory actions of 6 polyphenolic candidates in preventing IAPP and Aβ40 self-aggregation (Figure 5; Table 1) and showed that the polyphenol, EGCG, is the most effective candidate against the self-aggregation of both peptides. We then showed that EGCG was highly effective in preventing IAPP-Aβ40 co-aggregation at a kinetic, conformation and morphology levels (Figures 6–8) and in reducing its toxicity on PC-12 and RIN-m5F cells (Figure 9, Supplementary Figures S13, S14). To the best of our knowledge, this is the first study to report the inhibition of IAPP-Aβ40 co-aggregation by a polyphenolic small molecule and our current *in vitro* data holds significant impact in establishing a preventative therapy against the association between T2D and AD.

## Data Availability

The original contributions presented in the study are included in the article/Supplementary Material, further inquiries can be directed to the corresponding author.

## Abbreviations

AD, Alzheimer’s disease; Aβ, β-amyloid; EGCG, epigallocatechin gallate; HFIP, 1,1,1,3,3,3-Hexafluoro-2-propanol; IAPP, islet amyloid polypeptide; STEM, Scanning Transmission Electron Microscopy, ThT, Thioflavin-T; T2D, type two diabetes.
