# Supplementary material for: Linking Alzheimer’s Disease and Type 2 Diabetes: Characterization and Inhibition of Cytotoxic Aβ and IAPP Hetero-Aggregates
Source: Front Mol Biosci. 2022 Mar 17;9:842582. doi: 10.3389/fmolb.2022.842582 (PMC8968156; doi:10.3389/fmolb.2022.842582)
Supplement: Supplementary file 1 [file DataSheet1.PDF]

frontiers

Supplementary Material

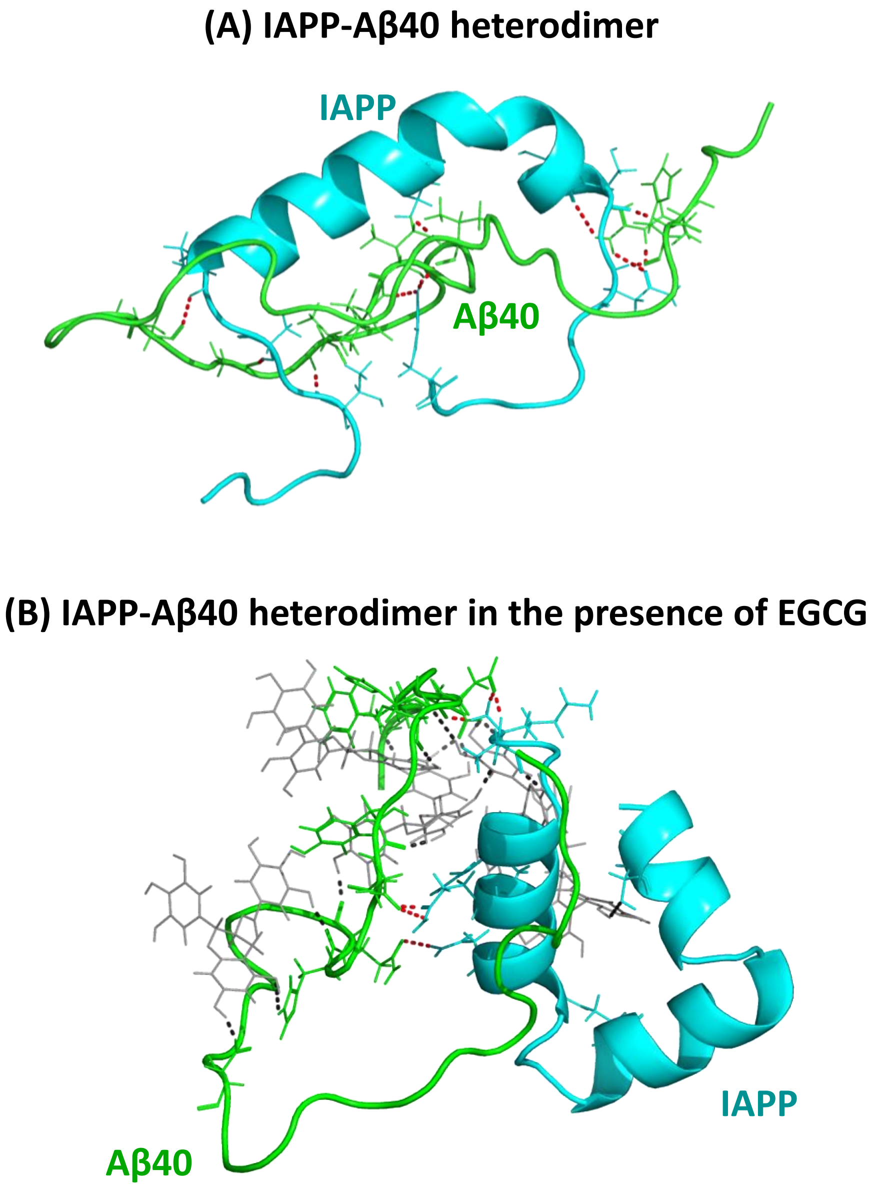

Supplementary Figure 1. Representative snapshots of IAPP-Aβ40 hetero-dimers in the absence (A) and presence of EGCG (B). Without EGCG, IAPP-Aβ40 hetero-dimer interface is stabilized by 10 inter-peptide hydrogen bonds (red dashed lines) that form between IAPP chain (cyan) and Aβ40 chain (green). The presence of 5 EGCG molecules (colored in grey) resulted in a smaller number of inter-peptide hydrogen bonds (red dashed lines) between IAPP and Aβ40 whereas inter-molecular hydrogen bonds (black dashed lines) were identified between each EGCG molecule and either IAPP or Aβ40 indicating that EGCG interaction with IAPP/Aβ40 interfere with the formation of a stable heterodimer interface. This figure was generated using PyMOL (Version 2).

```
Aβ   1 --D-AEFRHDS-GYEVHHQKLV-FFAEDV-GSNK-GAIIGLM---VGG-VV    40
         : |     : .  .  |:|. |.   | .||. |||  |.   ||. ..
IAPP 1 KCNTA-----TCA--T--QRLANFL---VHSSNNFGAI--LSSTNVGSNTY    37

Aβ (15-32)     QKLVFFAEDVGSNKGAII
               |:|: |    .:| |||:
IAPP (10-27)   QRLANFLVHSSNNFGAIL
```

Supplementary Figure 2. Global pairwise sequence alignment of Aβ40 and IAPP obtained using EMBOSS stretcher [1] reveals 25% identity and 31.4% similarity across their entire sequences. The two sub-sequences, Aβ40 (15-32) and IAPP (10-27), share a 38.9% identity and 55.6% similarity based on their local alignment obtained using LALIGN [2]. The vertical line (|) denotes identical residues, the colon (:) denotes residues with strongly similar properties and the period (.) denotes residues with weakly similar properties.

[1]Myers, E. W.; Miller, W. Optimal Alignments in Linear Space. Bioinformatics 1988, 4 (1), 11–17. https://doi.org/10.1093/bioinformatics/4.1.11.

[2]Huang, X.; Miller, W. A Time-Efficient, Linear-Space Local Similarity Algorithm. Advances in Applied Mathematics 1991, 12 (3), 337–357. https://doi.org/10.1016/0196-8858(91)90017-D.

Supplementary Table 1. Dimensions of the fibrillar and non-fibrillar assemblies observed by scanning transmission electron microscopy (STEM) for IAPP alone (20 µM and 40 µM), Aβ40 alone (20 µM and 40 µM) and the equimolar IAPP-Aβ40 samples at the indicated time-points during the self-aggregation and co-aggregation pathways.

**Sample**	**0-Hour**	**3-Hours**	**96-Hours**
Aβ40 (20µM)	Diameter: 15.5 ± 4.7 nm	Diameter: 37.4 ± 11 nm	Fibril diameter: 6.9 ± 1.4 nm
IAPP (20µM)	Diameter: 13.5 ± 3.8 nm	Fibril diameter: 10.7 ± 2.3 nm	Fibril diameter: 10.0 ± 2.1 nm
Aβ40 (40µM)	Diameter: 24.2 ± 5.2 nm	Fibril diameter: 5.2 ± 1.1 nm	Fibril diameter: 10.1 ± 1.2 nm
IAPP (40µM)	Diameter: 19.6 ± 6.2 nm	Fibril diameter: 12.1 ± 2.6 nm	Fibril diameter: 13.0 ± 2.5 nm
IAPP:Aβ40 (20µM:20µM)	Diameter: 38.4 ± 5.4 nm	Fibril diameter: 12.0 ± 2.2 nm	Fibril diameter: 9.5 ± 1.7 nm

IAPP:Aβ40 (20µM:20µM)
(3-Hours)

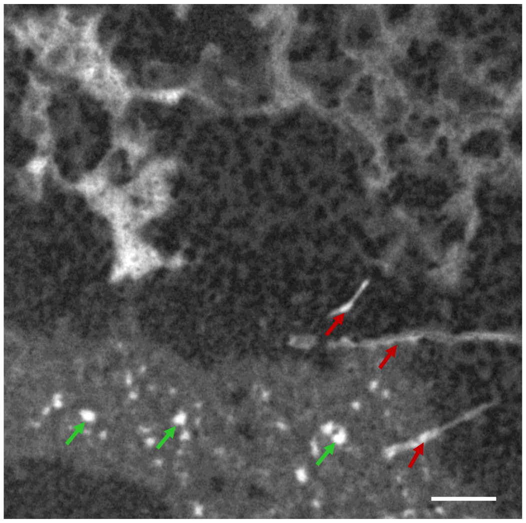

Supplementary Figure 3. STEM image of the 3 Hours-aged hetero-assemblies formed by the co-aggregation of IAPP-Aβ40 (20µM:20µM). In addition to Figure 2B, this STEM image demonstrates the formation of non-fibrillar (green arrows) and fibrillar (red arrows) structures in the IAPP-Aβ40 mixed sample. The scale bar represents 200 nm.

Supplementary Table 2. Deconvolution analysis of the CD spectra of fresh (0-Hour) and aggregated (96-Hours) samples containing IAPP alone, Aβ40 alone and equimolar IAPP-Aβ40.

Sample		α-helices	β-sheets	Turn	Unordered
0-Hour	**Aβ40 (10µM)**	8.3 %	9.9 %	32.0 %	49.8 %
	**IAPP (10µM)**	9.4 %	5.1 %	30.8 %	54.7 %
	**IAPP:Aβ40 (10µM:10µM)**	11.7 %	31.9 %	22.5 %	33.9 %
	**Aβ40 (20µM)**	5.3 %	27.5 %	25.0 %	42.2 %
	**IAPP (20µM)**	12.2 %	28.5 %	23.1 %	36.2 %
	**IAPP:Aβ40 (20µM:20µM)**	12.3 %	38.3 %	21.6 %	27.9 %
96-Hours	**Aβ40 (20µM)**	10.5 %	35.5 %	22.4 %	31.6 %
	**IAPP (20µM)**	10.1 %	39.4 %	21.3 %	29.2 %
	**IAPP:Aβ40 (20µM:20µM)**	1.1 %	53.3 %	21.8 %	23.9 %

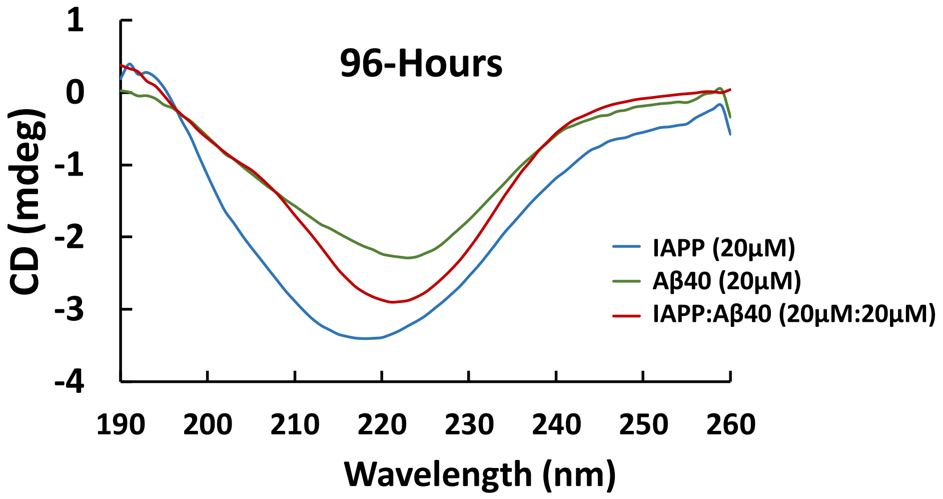

Supplementary Figure 4. Far-UV CD spectra of aged aggregates (96-Hours) of IAPP alone, Aβ40 alone and IAPP-Aβ40 showing pronounced peaks at 220 nm which indicate the formation of β-sheet-rich homo- or hetero- aggregates at the end of the incubation periods.

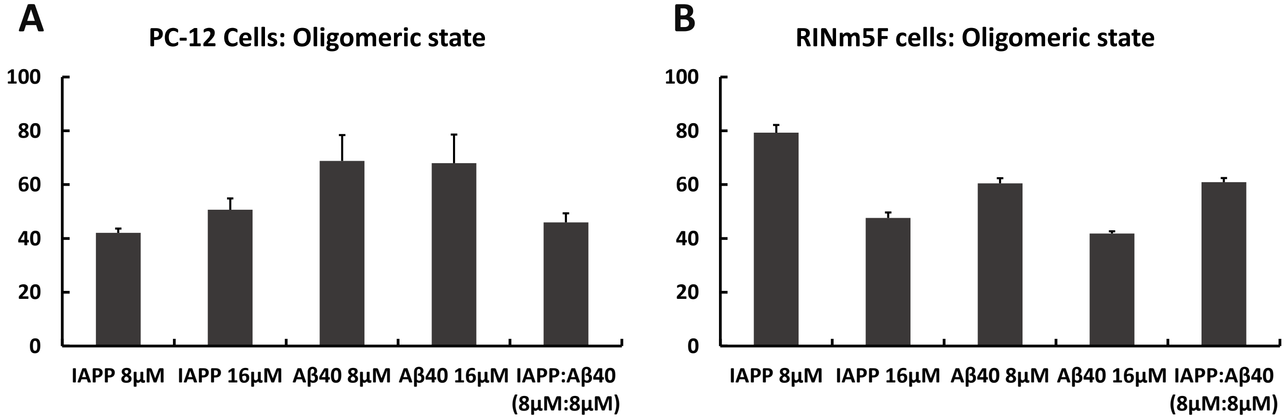

Supplementary Figure 5. MTT results showing the normalized cell viability rates of **(A)** PC-12 cells and **(B)** RIN-m5F cells after treatment with the 3 Hours-aged homo-oligomers (IAPP alone or Aβ40 alone) and hetero-oligomers (IAPP-Aβ40) at the indicated concentrations. Normalized cell viability rates were calculated relative to control samples (cells without peptide treatment). Data represent mean ± SE (n=3).

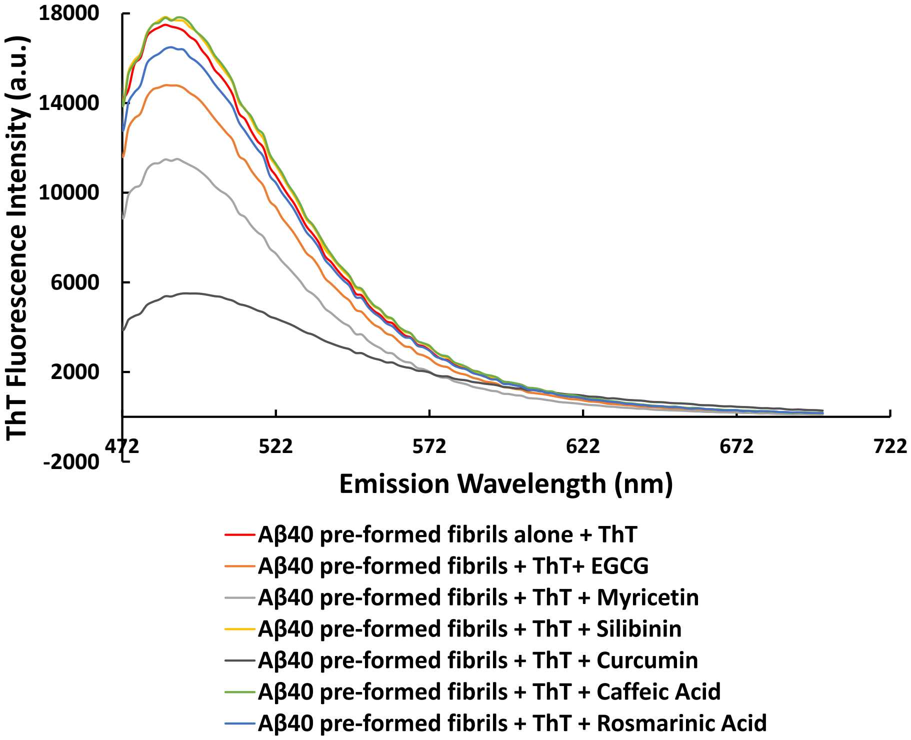

Supplementary Figure 6. ThT emission spectra (λ_{ex} 440 nm and λ_{em} 470 – 700 nm) were collected for samples containing a mixture of pre-formed Aβ40 fibrils and ThT (30 μM) in the absence and presence of each polyphenolic candidate. Each candidate was added at 40 μM to the mixture of pre-formed Aβ40 fibrils and ThT (30 μM) which was followed by the immediate measurements of the resulting ThT fluorescence emission spectra. The fluorescence values at λ_{em} 486 nm were utilized to measure the rates by which each candidate interferes with (i.e. reduces or increases) the control spectra the control spectra which is the ThT fluorescence of pre-formed Aβ40 fibrils and ThT (30μM) only. The interference rates are reported in Table 1.

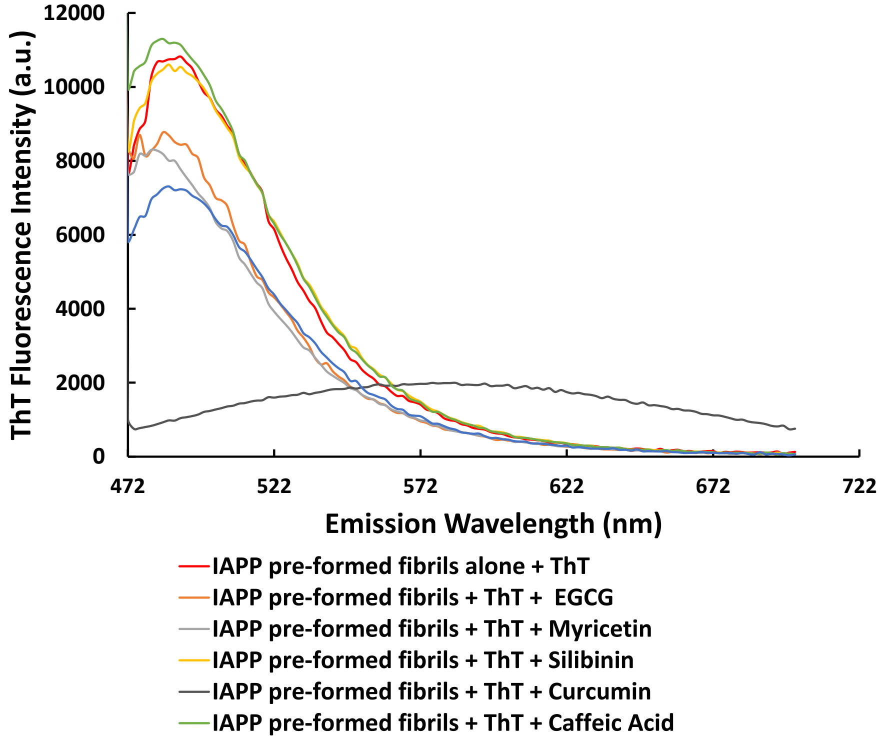

Supplementary Figure 7. ThT emission spectra (λ_{ex} 440 nm and λ_{em} 470 – 700 nm) were collected for samples containing a mixture of pre-formed IAPP fibrils and ThT (30 µM) in the absence and presence of each polyphenolic candidate. Each candidate was added at 40 µM to the mixture of pre-formed IAPP fibrils and ThT (30 µM) which was followed by the immediate measurements of the resulting ThT fluorescence emission spectra. The fluorescence values at λ_{em} 486 nm were utilized to measure the rates by which each candidate interferes with (i.e. reduces or increases) the control spectra which is the ThT fluorescence of pre-formed IAPP fibrils and ThT (30µM) only. The interference rates are reported in Table 1.

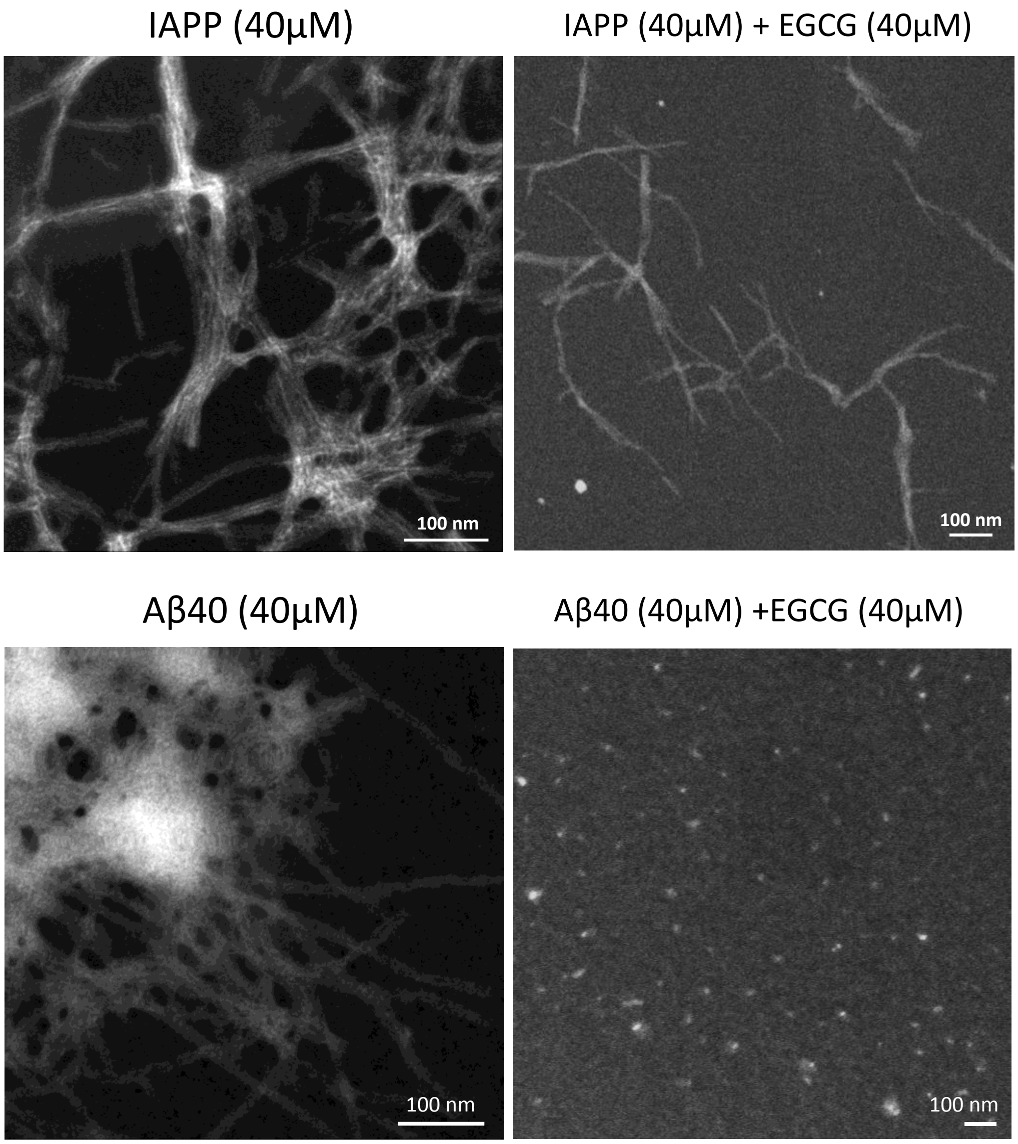

Supplementary Figure 8. Morphological characterization using STEM of the aged assemblies formed by incubating monomeric IAPP (40 µM) or Aβ40 (40 µM) in the absence and presence of an equimolar concentration of EGCG (40 µM).

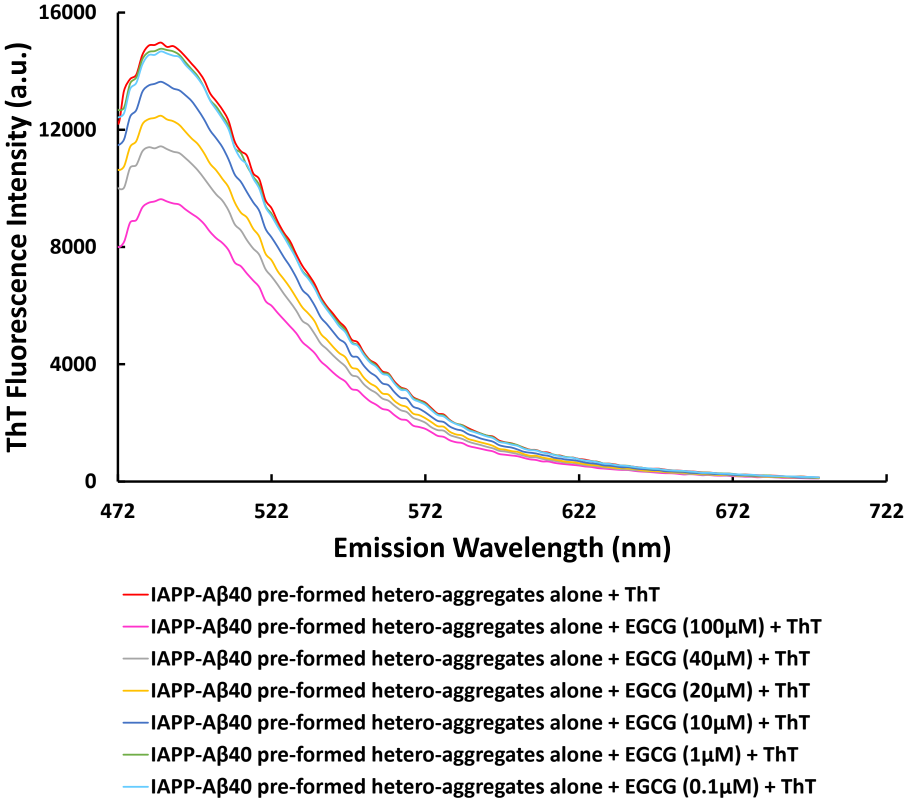

Supplementary Figure 9. ThT emission spectra (λ_{ex} 440 nm and λ_{em} 470 – 700 nm) were collected for samples containing a mixture of pre-formed IAPP-Aβ40 hetero-aggregates and ThT (30 µM) in the absence and presence of increasing EGCG concentrations. EGCG was added at the indicated concentrations to the mixture of pre-formed IAPP-Aβ40 hetero-aggregates and ThT (30 µM) which was followed by the immediate measurements of the resulting ThT fluorescence emission spectra. The fluorescence values at λ_{em} 486 nm were utilized to measure the rates by which EGCG interferes with (i.e. reduces) the control spectra which is the ThT fluorescence of pre-formed IAPP-Aβ40 hetero-aggregates and ThT (30µM) only. The interference rates are reported in Table 2.

Supplementary Table 3. Dimensions of the fibrillar and non-fibrillar assemblies observed by scanning transmission electron microscopy (STEM) for the equimolar IAPP-Aβ40 (20μM:20μM) samples in the absence and presence of increasing EGCG concentrations (10 μM – 100 μM) at indicated time-points during the co-aggregation pathways.

Sample	0-Hours	3-Hours	96-Hours
IAPP:Aβ40 (20μM:20μM)	Diameter of non-fibrillar aggregates: 38.4 ± 5.4 nm	Fibril diameter: 12.0 ± 2.2 nm	Fibril diameter: 9.5 ± 1.7 nm
IAPP:Aβ40 (20μM:20μM) + EGCG (10μM)	Diameter of non-fibrillar aggregates: 40.5 ± 14.9 nm	Diameter of non-fibrillar aggregates: 39.8 ± 9.3 nm	Fibril diameter: 14.7 ± 2.7 nm
IAPP:Aβ40 (20μM:20μM) + EGCG (20μM)	Diameter of non-fibrillar aggregates: 26.2 ± 6.3 nm	Diameter of non-fibrillar aggregates: 62.13 ± 10.6 nm	Fibril diameter: 14.5 ± 3.2 nm
IAPP:Aβ40 (20μM:20μM) + EGCG (40μM)	Diameter of non-fibrillar aggregates: 35.6 ± 12.9 nm	Diameter of non-fibrillar aggregates: 44.0 ± 5.2 nm	Fibril diameter: 10.7 ± 1.4 nm
IAPP:Aβ40 (20μM:20μM) + EGCG (100μM)	Diameter of non-fibrillar aggregates: 31.1 ± 9.3 nm	Diameter of non-fibrillar aggregates: 16.2 ± 4.7 nm	Fibril diameter: 10.3 ± 1.5 nm

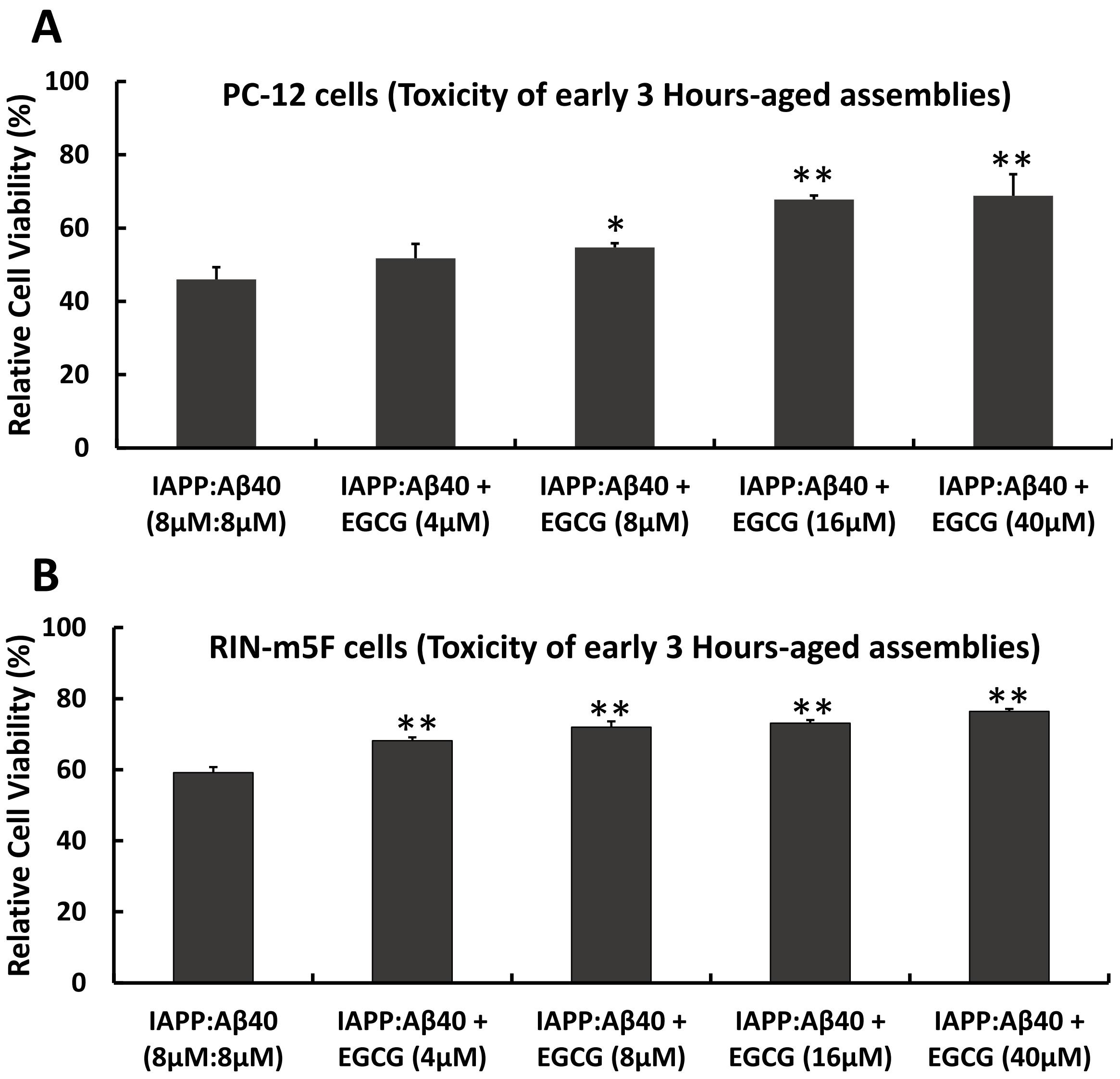

Supplementary Figure 10. MTT results showing the normalized cell viability rates of (A) PC-12 cells and (B) RIN-m5F cells after treatment with the 3 Hours aged hetero-aggregates (early aggregates) of IAPP-Aβ in the absence and presence of increasing EGCG concentrations. Samples at the 3-Hours time-point during the co-aggregation process were diluted in serum-free cell culture media before their addition to cells. The final concentration of IAPP-Aβ40 mixed sample was 8μM:8μM while the final EGCG concentrations ranged between 4 μM – 40 μM. Negative control or background values including the interference of EGCG with MTT only (no cells) were obtained from Figure S9, and subtracted from the absorbance values of cells treated with matching EGCG concentrations. Normalized cell viability rates were calculated relative to control samples (cells without the peptide or inhibitor treatment). Data represent mean ± SE (n=3). * p-value < 0.001, ** p-value < 0.0001.

Supplementary Table 4. Deconvolution analysis of the CD spectra of fresh (0-Hour) and aggregated (96-Hours) samples containing equimolar IAPP-Aβ40 (20µM:20µM) in the absence and presence of increasing EGCG concentrations (10µM – 100 µM).

Sample		α-helices	β-sheets	Turn	Unordered
**0-Hour**	**IAPP:Aβ40 (20µM:20µM)**	12.3 %	38.3 %	21.6 %	27.9 %
	**IAPP:Aβ40 (20µM:20µM) + EGCG (10µM)**	11.7 %	34.4 %	22.1 %	31.8 %
	**IAPP:Aβ40 (20µM:20µM) + EGCG (20µM)**	13.0 %	34.6 %	22.0 %	30.0 %
	**IAPP:Aβ40 (20µM:20µM) + EGCG (40µM)**	14.5 %	33.3 %	21.6 %	30.7 %
	**IAPP:Aβ40 (20µM:20µM) + EGCG (100µM)**	9.6 %	35.0 %	19.9 %	35.5 %
**96-Hours**	**IAPP:Aβ40 (20µM:20µM)**	1.1 %	53.3 %	21.8 %	23.9 %
	**IAPP:Aβ40 (20µM:20µM) + EGCG (10µM)**	3.5 %	48.6 %	19.8 %	28.0 %
	**IAPP:Aβ40 (20µM:20µM) + EGCG (20µM)**	7.6 %	45.1 %	26.0 %	21.7 %
	**IAPP:Aβ40 (20µM:20µM) + EGCG (40µM)**	9.7 %	33.9 %	24.1 %	32.3 %
	**IAPP:Aβ40 (20µM:20µM) + EGCG (100µM)**	5.4 %	34.4 %	23.2 %	37.0 %

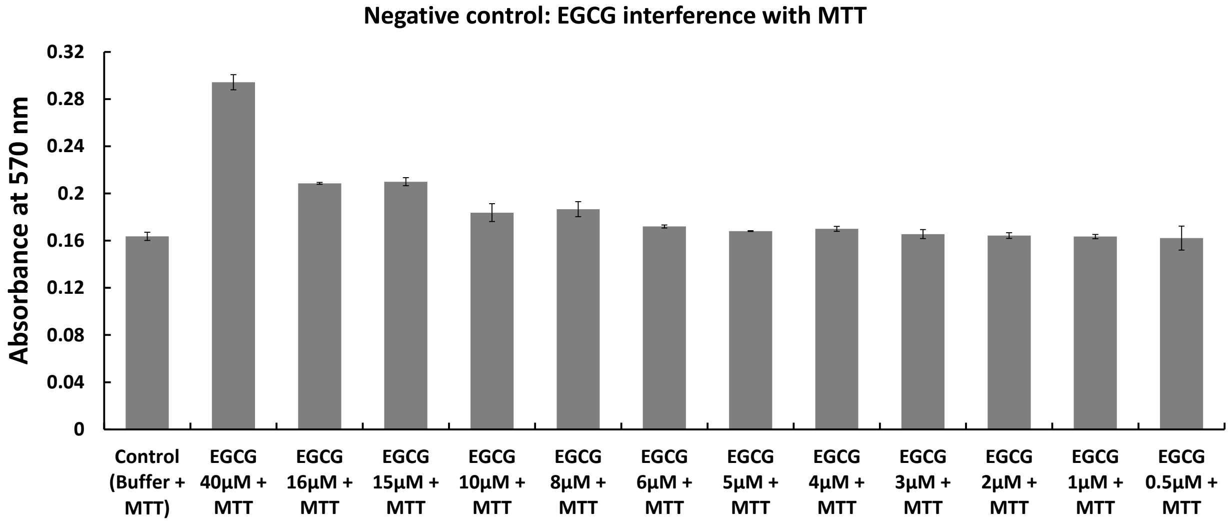

Supplementary Figure 11. Negative control tests for MTT assays were performed by measuring the absorbance at 570 nm of samples containing increasing EGCG concentration (0.5 µM – 40 µM) with MTT only (without cells). To simulate MTT experimental protocol, negative control samples were incubated with MTT for a duration of 4 hours in a humidified incubator (37 °C, 5% CO_2) before recording the absorbance values.

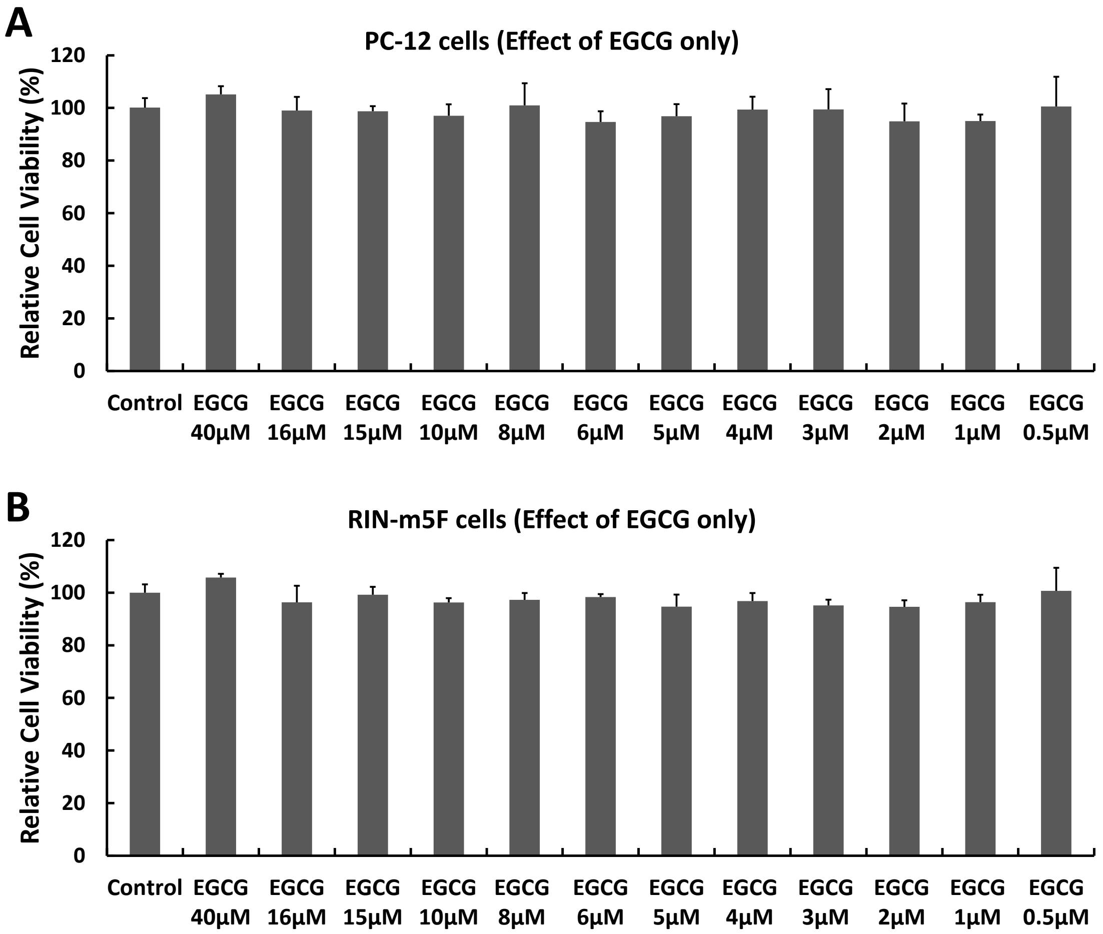

Supplementary Figure 12. PC-12 and RIN-m5F cell viability rates after treatment with EGCG at 10 µM – 100 µM without peptide addition. Data represent mean ± SE (n=3). Negative control or the background values of the interference of EGCG with MTT only (no cells) were obtained from Supplementary Figure 11, and subtracted from the absorbance values of cells treated with matching EGCG concentrations. Normalized cell viability rates were calculated relative to control samples (cells without the peptide or inhibitor treatment).

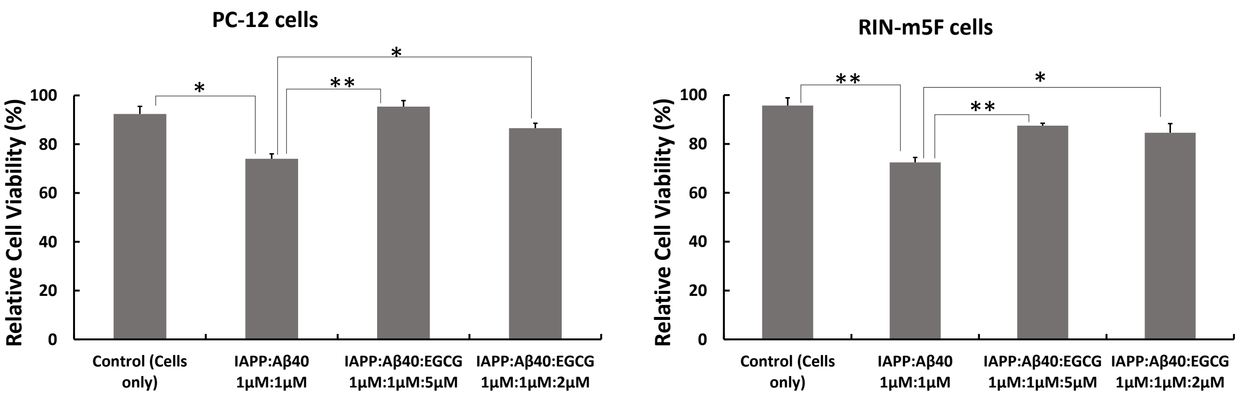

Supplementary Figure 13. Live/dead assay results showing the cell viability rates of (A) PC-12 cells and (B) RIN-m5F cells after treatment with 96 Hours-aged IAPP-Aβ40 hetero-aggregates (1µM:1µM) in the absence and presence of EGCG concentrations (2 µM and 5 µM). Cell viability rates were calculated as follows: (number of live cells/number of live cells + number of dead cells) x 100. ImageJ was used to count the number of live and dead cells. Data represent mean ± SE (n=3) from three independent images, * p-value < 0.001, ** p-value < 0.0005.

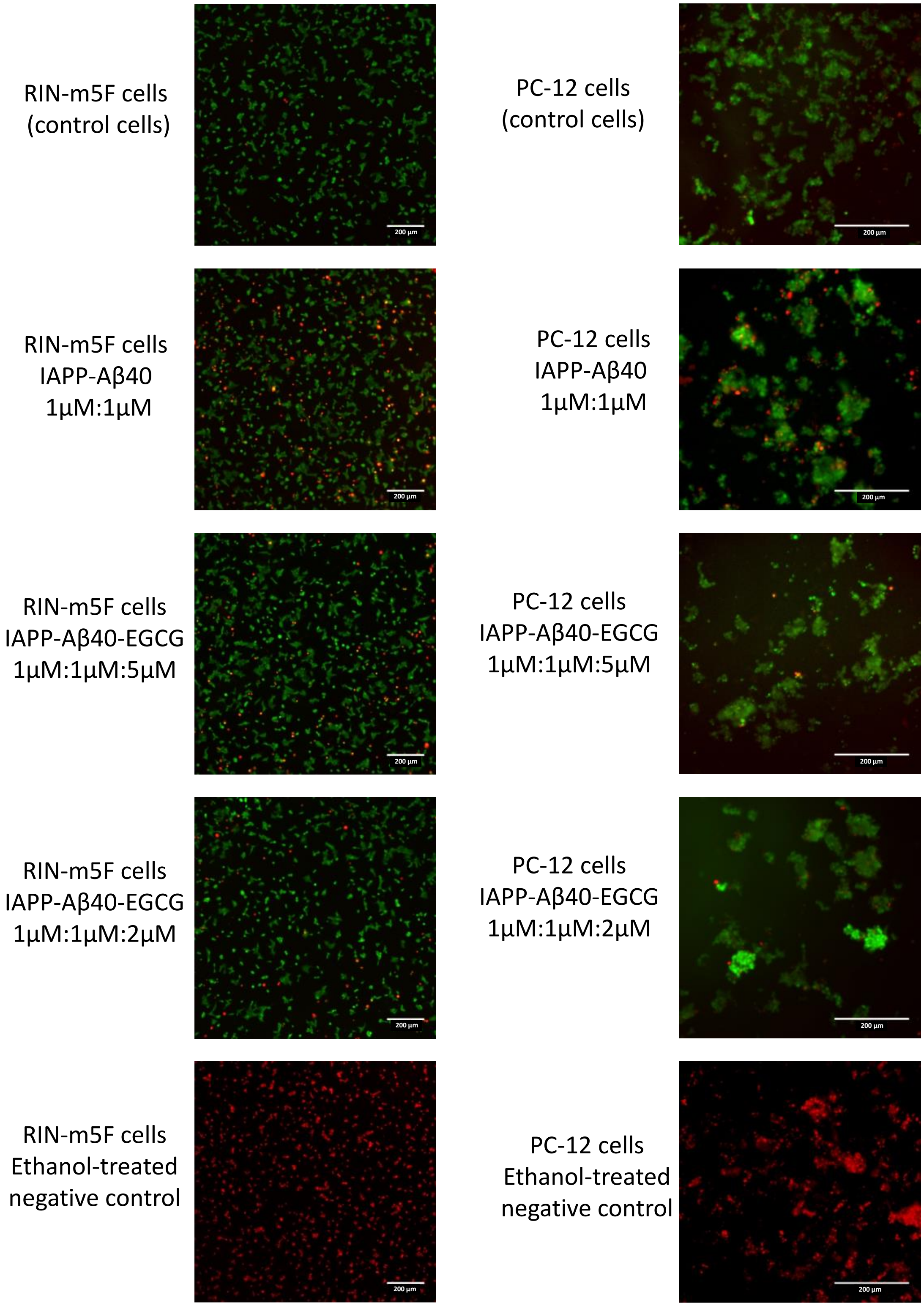

Supplementary Figure 14. Representative fluorescence images of PC-12 and RIN-m5F cells using the Live/Dead assay dyes (Calcein AM and BOBO-3 Iodide). Cells were treated with IAPP-Aβ40 (1µM:1µM) in the absence and presence of EGCG at 2 µM or 5 µM. All scale bars represent 200 µm.
